# Importance of genotype for risk stratification in arrhythmogenic right ventricular cardiomyopathy using the 2019 ARVC risk calculator^[Author-notes ehac235-FM1]^

**DOI:** 10.1093/eurheartj/ehac235

**Published:** 2022-06-29

**Authors:** Alexandros Protonotarios, Riccardo Bariani, Chiara Cappelletto, Menelaos Pavlou, Alba García-García, Alberto Cipriani, Ioannis Protonotarios, Adrian Rivas, Regitze Wittenberg, Maddalena Graziosi, Zafeirenia Xylouri, José M Larrañaga-Moreira, Antonio de Luca, Rudy Celeghin, Kalliopi Pilichou, Athanasios Bakalakos, Luis Rocha Lopes, Konstantinos Savvatis, Davide Stolfo, Matteo Dal Ferro, Marco Merlo, Cristina Basso, Javier Limeres Freire, Jose F Rodriguez-Palomares, Toru Kubo, Tomas Ripoll-Vera, Roberto Barriales-Villa, Loizos Antoniades, Jens Mogensen, Pablo Garcia-Pavia, Karim Wahbi, Elena Biagini, Aris Anastasakis, Adalena Tsatsopoulou, Esther Zorio, Juan R Gimeno, Jose Manuel Garcia-Pinilla, Petros Syrris, Gianfranco Sinagra, Barbara Bauce, Perry M Elliott

**Affiliations:** Institute of Cardiovascular Science, University College London, London, UK; Inherited Cardiovascular Disease Unit, St Bartholomew’s Hospital, London, UK; Department of Cardiac Thoracic, Vascular Sciences and Public Health, University of Padua, Padua, Italy; Cardio-Thoraco-Vascular Department, University of Trieste, Trieste, Italy; Department of Medicine, Karolinska Institutet, Stockholm, Sweden; Department of Statistical Science, University College London, London, UK; Inherited Cardiac Diseases Unit (CSUR-ERN), Department of Cardiology, Hospital Clínico Universitario Virgen de la Arrixaca, Murcia, Spain; Department of Cardiac Thoracic, Vascular Sciences and Public Health, University of Padua, Padua, Italy; Nikos Protonotarios Medical Centre, Naxos, Greece; Heart Failure and Inherited Cardiac Diseases Unit, Hospital Universitario Puerta de Hierro Majadahonda, Madrid, Spain; Department of Cardiology, Odense University Hospital, Odense, Denmark; Cardiology Unit, St Orsola Hospital, IRCCS Azienda Ospedaliero-Universitaria di Bologna, Bologna, Italy; Nikos Protonotarios Medical Centre, Naxos, Greece; Unidad de Cardiopatías Familiares, Instituto de Investigación Biomédica de A Coruña (INIBIC), Complexo Hospitalario Universitario de A Coruña, Servizo Galego de Saúde (SERGAS), Universidade da Coruña, CIBERCV, A Coruña, Spain; Cardio-Thoraco-Vascular Department, University of Trieste, Trieste, Italy; Department of Cardiac Thoracic, Vascular Sciences and Public Health, University of Padua, Padua, Italy; Department of Cardiac Thoracic, Vascular Sciences and Public Health, University of Padua, Padua, Italy; Institute of Cardiovascular Science, University College London, London, UK; Inherited Cardiovascular Disease Unit, St Bartholomew’s Hospital, London, UK; Institute of Cardiovascular Science, University College London, London, UK; Inherited Cardiovascular Disease Unit, St Bartholomew’s Hospital, London, UK; European Reference Networks for rare, low prevalence and complex diseases of the heart (ERN GUARD-Heart); Institute of Cardiovascular Science, University College London, London, UK; Inherited Cardiovascular Disease Unit, St Bartholomew’s Hospital, London, UK; European Reference Networks for rare, low prevalence and complex diseases of the heart (ERN GUARD-Heart); Cardio-Thoraco-Vascular Department, University of Trieste, Trieste, Italy; Department of Medicine, Karolinska Institutet, Stockholm, Sweden; Cardio-Thoraco-Vascular Department, University of Trieste, Trieste, Italy; Cardio-Thoraco-Vascular Department, University of Trieste, Trieste, Italy; Department of Cardiac Thoracic, Vascular Sciences and Public Health, University of Padua, Padua, Italy; European Reference Networks for rare, low prevalence and complex diseases of the heart (ERN GUARD-Heart); Unidad de Cardiopatías Familiares, Servicio de Cardiología, Hospital Universitario Vall d'Hebron, Vall d'Hebron Institut de Recerca (VHIR), Universitat Autonoma de Barcelona, Barcelona, Spain; Centre for Biomedical Network Research on Cardiovascular Diseases (CIBERCV), Madrid, Spain; European Reference Networks for rare, low prevalence and complex diseases of the heart (ERN GUARD-Heart); Unidad de Cardiopatías Familiares, Servicio de Cardiología, Hospital Universitario Vall d'Hebron, Vall d'Hebron Institut de Recerca (VHIR), Universitat Autonoma de Barcelona, Barcelona, Spain; Centre for Biomedical Network Research on Cardiovascular Diseases (CIBERCV), Madrid, Spain; Department of Cardiology and Geriatrics, Kochi Medical School, Kochi University, Japan; Inherited Cardiovascular Diseases Unit, Son Llatzer University Hospital & IdISBa, Palma de Mallorca, Spain; Unidad de Cardiopatías Familiares, Instituto de Investigación Biomédica de A Coruña (INIBIC), Complexo Hospitalario Universitario de A Coruña, Servizo Galego de Saúde (SERGAS), Universidade da Coruña, CIBERCV, A Coruña, Spain; European Reference Networks for rare, low prevalence and complex diseases of the heart (ERN GUARD-Heart); Cyprus Institute of Cardiomyopathies and Inherited Cardiovascular Diseases, Nicosia, Cyprus; Aalborg University Hospital, Denmark; Heart Failure and Inherited Cardiac Diseases Unit, Hospital Universitario Puerta de Hierro Majadahonda, Madrid, Spain; European Reference Networks for rare, low prevalence and complex diseases of the heart (ERN GUARD-Heart); Centre for Biomedical Network Research on Cardiovascular Diseases (CIBERCV), Madrid, Spain; Cardiology Department, AP-HP, Cochin Hospital, FILNEMUS, Centre de Référence de Pathologie Neuromusculaire Nord/Est/Île-de-France, Paris-Descartes, Sorbonne Paris Cité University, Paris, France; Cardiology Unit, St Orsola Hospital, IRCCS Azienda Ospedaliero-Universitaria di Bologna, Bologna, Italy; Unit of Inherited and Rare Cardiovascular Diseases, Onassis Cardiac Surgery Centre, Athens, Greece; Nikos Protonotarios Medical Centre, Naxos, Greece; Unit of Inherited and Rare Cardiovascular Diseases, Onassis Cardiac Surgery Centre, Athens, Greece; Centre for Biomedical Network Research on Cardiovascular Diseases (CIBERCV), Madrid, Spain; Inherited Cardiac Diseases and Sudden Death Unit, Department of Cardiology, Hospital Universitario y Politécnico La Fe, CaFaMuSMe Research Group, Instituto de Investigación Sanitaria La Fe, Valencia, Spain; Inherited Cardiac Diseases Unit (CSUR-ERN), Department of Cardiology, Hospital Clínico Universitario Virgen de la Arrixaca, Murcia, Spain; European Reference Networks for rare, low prevalence and complex diseases of the heart (ERN GUARD-Heart); Centre for Biomedical Network Research on Cardiovascular Diseases (CIBERCV), Madrid, Spain; Centre for Biomedical Network Research on Cardiovascular Diseases (CIBERCV), Madrid, Spain; Heart Failure and Familial Heart Diseases Unit, Cardiology Service, Hospital Universitario Virgen de la Victoria, IBIMA, Málaga, Spain; Institute of Cardiovascular Science, University College London, London, UK; Cardio-Thoraco-Vascular Department, University of Trieste, Trieste, Italy; Department of Cardiac Thoracic, Vascular Sciences and Public Health, University of Padua, Padua, Italy; Institute of Cardiovascular Science, University College London, London, UK; Inherited Cardiovascular Disease Unit, St Bartholomew’s Hospital, London, UK; European Reference Networks for rare, low prevalence and complex diseases of the heart (ERN GUARD-Heart)

**Keywords:** Arrhythmogenic right ventricular cardiomyopathy, Sudden cardiac death, Ventricular arrhythmia, Risk stratification, Genotype

## Abstract

**Aims:**

To study the impact of genotype on the performance of the 2019 risk model for arrhythmogenic right ventricular cardiomyopathy (ARVC).

**Methods and results:**

The study cohort comprised 554 patients with a definite diagnosis of ARVC and no history of sustained ventricular arrhythmia (VA). During a median follow-up of 6.0 (3.1,12.5) years, 100 patients (18%) experienced the primary VA outcome (sustained ventricular tachycardia, appropriate implantable cardioverter defibrillator intervention, aborted sudden cardiac arrest, or sudden cardiac death) corresponding to an annual event rate of 2.6% [95% confidence interval (CI) 1.9–3.3]. Risk estimates for VA using the 2019 ARVC risk model showed reasonable discriminative ability but with overestimation of risk. The ARVC risk model was compared in four gene groups: *PKP2* (*n* = 118, 21%); desmoplakin (*DSP*) (*n* = 79, 14%); other desmosomal (*n* = 59, 11%); and gene elusive (*n* = 160, 29%). Discrimination and calibration were highest for *PKP2* and lowest for the gene-elusive group. Univariable analyses revealed the variable performance of individual clinical risk markers in the different gene groups, e.g. right ventricular dimensions and systolic function are significant risk markers in *PKP2* but not in *DSP* patients and the opposite is true for left ventricular systolic function.

**Conclusion:**

The 2019 ARVC risk model performs reasonably well in gene-positive ARVC (particularly for *PKP2*) but is more limited in gene-elusive patients. Genotype should be included in future risk models for ARVC.

## Structured Graphical Abstract

**Structured Graphical Abstract:**
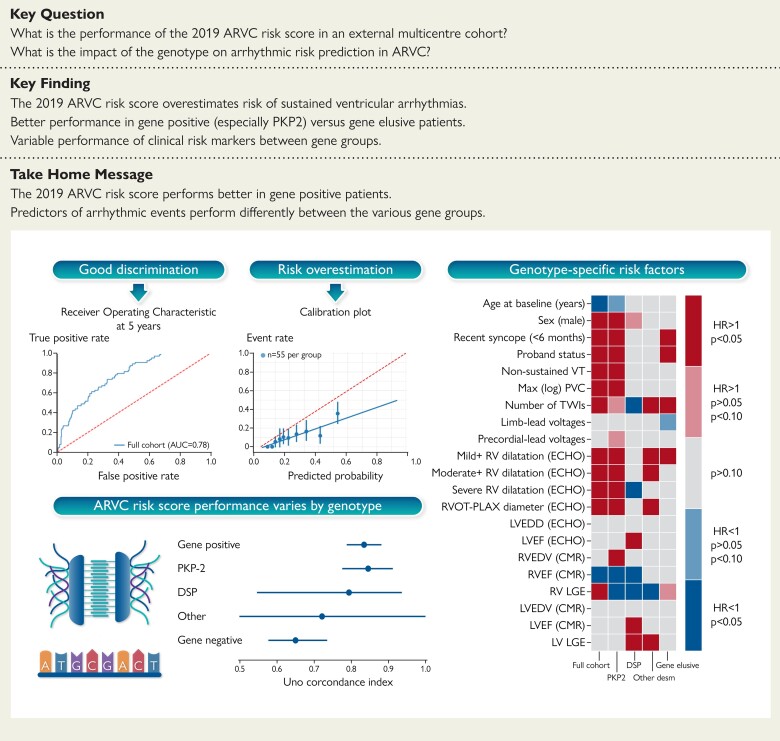
ARVC, arrhythmogenic right ventricular cardiomyopathy; AUC, area under the curve; ECHO, echocardiography; CMR, cardiac magnetic resonance; HR, hazard ratio; LGE, late gadolinium enhancement; LV, left ventricular; LVEDD, left ventricular enddiastolic diameter; LVEF, left ventricular ejection fraction; PLAX, parasternal long-axis; PVC, premature ventricular complex; RV, right ventricular; RVEDV, right ventricular end-diastolic volume; RVEF, right ventricular ejection fraction; RVOT, right ventricular outflow tract; TWI, T-wave inversion; VT, ventricular tachycardia.

**See the editorial comment for this article ‘Arrhythmogenic right ventricular cardiomyopathy: the never-ending quest for a risk calculator’, by Estelle Gandjbakhch and Annina S. Vischer, https://doi.org/10.1093/eurheartj/ehac324.**

## Introduction

Arrhythmogenic right ventricular cardiomyopathy (ARVC) is a heritable heart muscle disorder characterized by ventricular arrhythmia (VA) that can lead to sudden cardiac death (SCD).^1^ The clinical diagnosis of ARVC is based on consensus criteria that have been developed to capture the right ventricular abnormalities typically manifested by patients.^2^ The genetic architecture of ARVC is diverse, with pathogenic variants first described in genes coding for desmosomal proteins and then, more recently, in genes encoding a number of non-desmosomal proteins.^3^ Although limited, current data suggest that clinical outcomes may differ between genotypes.^4–6^

Implantable cardioverter defibrillator (ICD) therapy in patients with ARVC and documented sustained VA is associated with improved survival^7,8^ and is considered a Class 1 indication in current practice guidelines. However, the indications for primary prevention ICDs in individuals with ARVC that have no history of sustained VA are less certain and until recently were based on a subjective evaluation of clinical risk markers supported by evidence of variable quality.^9^ In a recent landmark study, a risk tool designed to provide individualized risk estimates was proposed^10^ but the model was agnostic to the underlying genetic cause of the disease.

In this study, we hypothesized that disease aetiology influences the risk of VA in ARVC and sought to determine the performance of the 2019 ARVC risk model in a large multicentre cohort of patients stratified by genotype. During the review of this paper, a correction to the 2019 ARVC risk model was published.^11^ Our analysis is based on the corrected risk score but data on the original model are presented in the Supplementary material online to illustrate the impact of the correction on its performance.

## Methods

### Study design and participating centres

This is an international, multicentre retrospective observational cohort study using data on consecutively evaluated patients with ARVC recruited from 17 centres in 7 countries (see Supplementary material online, *Table S1*). All participating centres specialize in the clinical management of cardiomyopathy patients. The study conforms to the Declaration of Helsinki and all centres have local ethical approval.

### Study population

Patients were enrolled according to pre-specified inclusion criteria. Specifically: (i) a definite diagnosis of ARVC according to the 2010 task-force criteria (TFC)^2^; (ii) no history of sustained VA before first assessment at the participating centre; (iii) a follow-up period of at least 1 month; and (iv) age of diagnosis of 14 years or more.

### Data collection and study variables

Study data were collected independently by each centre and managed using REDCap (Research Electronic Data Capture) electronic data capture tools hosted at University College London.^12^ Standard data collection procedures, in accordance with general data protection regulation, were followed (see Supplementary material online, *Table S2*). Data were collected at each centre following review of medical and death records using variables derived from those used by Cadrin-Tourigny *et al*.^10^ The time of diagnosis was set as the baseline timepoint. The baseline phenotypic data comprised the primary dataset used for most analyses. As not all individuals had cardiac magnetic resonance (CMR) imaging at their baseline timepoint, we collected a second dataset of all the phenotypic data at the time of a first CMR if performed during follow-up (CMR dataset). The latter was used only for data imputation and sensitivity analysis purposes (see Supplementary material online).

### Genetic analysis

Clinical genetic testing is performed routinely at all participating centres using next-generation sequencing or direct sequencing of candidate genes associated with ARVC.^3^ In all probands, genes that have been shown to have a moderate or strong association with ARVC were analysed^3^: plakophilin-2 (*PKP2*), desmoplakin (*DSP*), plakoglobin (*JUP*), desmoglein-2 (*DSG2*), desmocollin-2 (*DSC2*), transmembrane protein 43 (*TMEM43*), desmin (*DES*), and phospholamban (*PLN*). Genetic variants were classified according to the American College of Medical Genetics and Genomics guidelines following independent review (P.S. and A.P.).^13^ Where additional evidence of pathogenicity (e.g. segregation data) for novel variants was available from the contributing centre, they were re-classified accordingly. A list of all the identified variants and their respective classification are reported in Supplementary material online, *Table S3*. Gene-elusive and gene-positive genetic status was defined according to the absence or presence of a pathogenic or likely pathogenic variant in any of the genes tested in each patient.

### Outcomes

The primary outcome of this study was the first VA during follow-up and was a composite of: (i) spontaneous sustained ventricular tachycardia (VT), defined as VT lasting ≥30 s or with haemodynamic compromise at ≥100 b.p.m. or terminated by electrical cardioversion; (ii) ICD intervention, defined as ICD shock or anti-tachycardia overdrive pacing delivered in response to a ventricular tachyarrhythmia confirmed by intracardiac ECG data; (iii) SCD, defined as death of cardiac origin that occurred unexpectedly within 1 h of the onset of new symptoms or a death that was unwitnessed and unexpected; (iv) aborted sudden cardiac arrest, defined as SCD, that is reversed by cardiopulmonary resuscitation and/or defibrillation or cardioversion. Death from any other cause and heart transplantation was also recorded.

### Predictors

Potential predictors similar to those used by Cadrin-Tourigny *et al*.^10^ were studied. All predictor variables were determined at the baseline timepoint or within 1 year of baseline but always before the arrhythmic outcome. Recent syncope was defined as cardiac syncope during the last 6 months before baseline.

### General statistical methods

All data manipulation and analyses were performed using the Python programming language (Version 3.8, Python Software Foundation, https://www.python.org/). Continuous variables were tested for normality of distribution by visual inspection of histograms and statistical normality tests (Shapiro–Wilk). Normally distributed variables are expressed as mean ± SD and non-normally distributed variables as median (25th, 75th percentiles). Categorical variables are reported as counts and percentages, as appropriate. The *TableOne* and *Scipy* libraries were used for the construction of summary statistics tables and for all comparisons.^14^ The *Seaborn* and *Matplotlib* libraries were utilized for data visualization.^15^ The *zEpid* library was used to calculate incidence rates.^16^

Follow-up time was calculated as the difference in age between the baseline (specific to each dataset) and the age when the study endpoint or censoring was reached. The annual event rate was calculated by dividing the number of patients reaching the endpoint by the total follow-up period for that endpoint. The cumulative probability for the occurrence of an outcome was estimated using the Aalen-Johansen estimate in order to take into account competing risks.^17,18^ Competing events were defined as the occurrence of heart transplantation or non-arrhythmic death. The *Lifelines* library was used for all time-to-event analyses.^19^ Fine-Gray regression was used to model the impact of clinical predictors on the arrhythmic outcome, in the context of competing risks.^20^ Fine-Gray regression was performed through the *cmprsk* library from the R-project through an R to Python interface.^21^ Hazard ratios and 95% confidence intervals (CIs) were reported. Bonferroni correction was used to correct *P*-values when multiple comparisons were made. *P*-values < 0.05 were considered significant.

### Model validation

The corrected 5-year ARVC risk score was calculated according to the proposed formula^10,11^:

$$P(\mathrm{VA})=1-S_{o}{\left(t\right)}^{exp(LP)}$$

where *S_o_*(*t*) is the baseline survival probability at the time *t*, which is 0.8396 at the 5-year mark (*t* = 5). The linear predictor was calculated as 0.488 × sex − 0.022 × age + 0.657 × history of recent cardiac syncope + 0.811 × history of NSVT + 0.170 × ln(24 h PVC count) + 0.113 × sum of anterior and inferior leads with TWI − 0.025 × RVEF

where NSVT is non-sustained ventricular tachycardia; PVC, premature ventricular complex; TWI, T-wave inversion; RVEF, right ventricular ejection fraction.

Binary parameters (male sex, history of recent cardiac syncope, and history of NSVT) were considered as 1 = positive and 0 = negative. The original 5-year ARVC risk score differs in regard to the *S_o_*(*t*) parameter (see Supplementary material online).

Model validation has been developed according to standard practices, assessing both discrimination and calibration.^22^ The discriminatory performance of the model was assessed using the Uno concordance index as obtained by the *sksurv.metrics.concordance_index_ipcw* function.^23^ Due to the time dependency of the outcomes, we also opted to use a time-dependent receiver operating characteristic curve analyses for the 5-year ARVC risk score.^24^ The *sksurv.metrics.roc_auc_score* function was used to calculate the time-dependent area under the curve at 5 years. In order to assess the model’s calibration, calibration plots were constructed using the *sklearn.calibration.calibration_curve* and *seaborn.regplot* functions.^25^ Bins of equal number of patients were created. All the model-validation analyses were repeated for each gene group. 95% CIs were obtained using a bootstrap procedure with 10 000 iterations of random sampling with replacement.

### Missing data

Missing data were addressed for the model-validation analyses. The *Missingno* library was utilized to visualize missing data (Supplementary material online, *Methods and Figure S1* ).^26^ Missing data were assumed to be missing at random and were imputed using the multiple imputation with chained equations method.^27^ The *Sklearn* library (*impute.IterativeImputer*) was utilized to perform data imputation.^28^ A total of 10 imputation rounds were performed before returning the imputations computed during the final round. A round is a single imputation of each feature with missing values. Sensitivity analyses were performed (Supplementary material online, *Methods*).

## Results

A total of 554 patients were enrolled from 17 centres. Demographic, genetic, clinical, outcome characteristics of patients and missing data in either dataset are reported in *Table 1*.

**Table 1:** Demographic, genetic, and clinical characteristics of patients according to the occurrence of the primary endpoint

	NA	Overall	No VA	VA	*P*-value
		(*n* = 554)	(*n* = 454)	(*n* = 100)	
**Baseline**					
Age (years)	0	41.0 (27.2,53.1)	42.8 (29.0,54.0)	37.0 (21.0,49.0)	0.002
Male sex	0	302 (54.5)	231 (50.9)	71 (71.0)	<0.001
Genotype	0				0.136
Gene elusive		157 (28.3)	138 (30.4)	19 (19.0)	
DES		5 (0.9)	4 (0.9)	1 (1.0)	
DSC2		11 (2.0)	9 (2.0)	2 (2.0)	
DSG2		27 (4.9)	24 (5.3)	3 (3.0)	
DSP		79 (14.3)	68 (15.0)	11 (11.0)	
FLNC		10 (1.8)	10 (2.2)		
JUP		21 (3.8)	16 (3.5)	5 (5.0)	
Multiple		13 (2.3)	8 (1.8)	5 (5.0)	
Not performed		104 (18.8)	79 (17.4)	25 (25.0)	
PKP2		118 (21.3)	91 (20.0)	27 (27.0)	
PLN		3 (0.5)	2 (0.4)	1 (1.0)	
RBM20		1 (0.2)	1 (0.2)		
TMEM43		2 (0.4)	1 (0.2)	1 (1.0)	
Ethnicity	1				0.476
Caucasian		518 (93.7)	427 (94.3)	91 (91.0)	
African		15 (2.7)	11 (2.4)	4 (4.0)	
Asian		20 (3.6)	15 (3.3)	5 (5.0)	
Proband status	0	304 (54.9)	234 (51.5)	70 (70.0)	0.001
Any symptoms	0	265 (47.8)	202 (44.5)	63 (63.0)	0.001
Recent syncope	0	32 (5.8)	17 (3.7)	15 (15.0)	<0.001
Structural TFC—minor	0	72 (13.0)	62 (13.7)	10 (10.0)	0.002
Structural TFC—major		290 (52.3)	222 (48.9)	68 (68.0)	
Tissue TFC—minor	0	11 (2.0)	10 (2.2)	1 (1.0)	0.693
Tissue TFC—major		29 (5.2)	23 (5.1)	6 (6.0)	
Repolarization TFC—minor	0	91 (16.4)	78 (17.2)	13 (13.0)	0.017
Repolarization TFC—major		261 (47.1)	201 (44.3)	60 (60.0)	
Depolarization TFC—minor	0	198 (35.7)	169 (37.2)	29 (29.0)	0.132
Depolarization TFC—major		58 (10.5)	43 (9.5)	15 (15.0)	
Arrhythmia TFC—minor	0	322 (58.1)	266 (58.6)	56 (56.0)	<0.001
Arrhythmia TFC—major		96 (17.3)	60 (13.2)	36 (36.0)	
Family history TFC—minor	0	41 (7.4)	36 (7.9)	5 (5.0)	0.024
Family history TFC—major		367 (66.2)	309 (68.1)	58 (58.0)	
RBBB	35				0.206
None		433 (83.4)	355 (83.3)	78 (83.9)	
iRBBB		41 (7.9)	37 (8.7)	4 (4.3)	
cRBBB		45 (8.7)	34 (8.0)	11 (11.8)	
LBBB	42				0.062
None		477 (93.2)	393 (93.3)	84 (92.3)	
LAFB		13 (2.5)	13 (3.1)		
LPFB		2 (0.4)	1 (0.2)	1 (1.1)	
LBBB		12 (2.3)	7 (1.7)	5 (5.5)	
IVCD		5 (1.0)	5 (1.2)		
QRS duration (ms)	148	100.0 (90.0,112.0)	100.0 (90.0,110.0)	100.0 (90.0,120.0)	0.306
TAD (ms)	217	52.0 (40.0,60.0)	52.0 (40.0,60.0)	55.0 (40.0,60.0)	0.787
Epsilon waves	0	51 (11.3)	34 (9.3)	17 (19.5)	
Number of TWIs	0	3.0 (1.0,4.0)	3.0 (1.0,4.0)	4.0 (2.0,5.0)	<0.001
Limb-lead voltages (mV)	213	0.9 (0.6,1.2)	0.9 (0.6,1.2)	0.8 (0.5,1.2)	0.486
Precordial-lead voltages (mV)	219	1.5 (1.0,2.0)	1.5 (1.0,2.0)	1.4 (1.0,2.1)	0.616
PVCs (per 24 h)	77	1232.0 (382,3044)	1126.5 (273,3022)	1695.0 (983,3315)	0.004
Non-sustained VT	0	234 (42.2)	176 (38.8)	58 (58.0)	0.001
RV dilatation (ECHO)	41				<0.001
None		212 (41.3)	192 (45.9)	20 (21.1)	
Mild		134 (26.1)	108 (25.8)	26 (27.4)	
Moderate		103 (20.1)	79 (18.9)	24 (25.3)	
Severe		64 (12.5)	39 (9.3)	25 (26.3)	
RVOT-PLAX diameter (ECHO)	249	33.0 (29.0,37.0)	32.0 (28.0,36.8)	36.0 (32.0,39.0)	0.001
LVEDD (ECHO)	176	50.0 (47.0,54.8)	50.0 (47.0,54.2)	50.0 (46.0,54.8)	0.664
LVEF (ECHO)	23	57.5 (50.0,65.0)	57.0 (50.0,65.0)	58.5 (51.0,65.0)	0.159
RVEDV (CMR)	281	181 (140,219)	178 (137,212)	205 (168,239)	0.008
RVEF (CMR)	263	46.0 (39.0,55.0)	48.0 (41.0,56.0)	39.0 (35.0,45.0)	<0.001
RV LGE	247	113 (36.8)	87 (33.5)	26 (55.3)	0.007
LVEDV (CMR)	265	154 (126,186)	156 (128,186)	145 (107,170)	0.1
LVEF (CMR)	253	57.0 (48.0,63.0)	56.5 (47.0,63.0)	58.0 (52.0,62.5)	0.308
LV LGE	247	176 (57.3)	150 (57.9)	26 (54.2)	0.746
Anti-arrhythmic at baseline	1				<0.001
None		425 (76.9)	365 (80.4)	60 (60.6)	
Amiodarone		35 (6.3)	25 (5.5)	10 (10.1)	
Sotalol		64 (11.6)	46 (10.1)	18 (18.2)	
Class IC		10 (1.8)	5 (1.1)	5 (5.1)	
Mexiletine		2 (0.4)		2 (2.0)	
Beta-blocker (excluding sotalol) at baseline, *n* (%)	1	213 (38.5)	175 (38.5)	38 (38.4)	0.933
**Follow-up**					
Follow-up duration (years)	0	6.0 (3.1,12.5)	5.9 (3.0,11.6)	9.2 (5.1,14.5)	0.001
ICD implantation	0	263 (47.5)	185 (40.7)	78 (78.0)	<0.001
Heart transplantation	0	17 (3.1)	13 (2.9)	4 (4.0)	0.525
Overall mortality	0	43 (7.8)	24 (5.3)	19 (19.0)	<0.001
VT ablation	0	21 (3.8)	5 (1.1)	16 (16.0)	<0.001
Anti-arrhythmic at follow-up	1				<0.001
None		380 (68.7)	336 (74.0)	44 (44.4)	
Amiodarone		49 (8.9)	27 (5.9)	22 (22.2)	
Sotalol		101 (18.3)	75 (16.5)	26 (26.3)	
Class IC		8 (1.4)	4 (0.9)	4 (4.0)	
Mexiletine		1 (0.2)	1 (0.2)		
Beta-blocker (excluding sotalol) at last follow-up, *n* (%)	1	266 (48.1)	218 (48.0)	48 (48.5)	0.979

Categorical variables are reported as *n* (%). Continuous variables are reported as mean ± SD or median (25th, 75th percentiles) according to the distribution normality. CMR, cardiac magnetic resonance; cRBBB, complete right bundle branch block; ECHO, echocardiography; ICD, implantable cardioverter defibrillator; iRBBB, incomplete right bundle branch block; IVCD, intraventricular conduction delay; LAFB, left anterior fascicular block; LBBB, left bundle branch block; LGE, late gadolinium enhancement; LPFB, left posterior fascicular block; LV, left ventricle; LVEDD, left ventricular end-diastolic diameter; LVEDV, left ventricular end-diastolic volume; LVEF, left ventricular ejection fraction; NA, not available; PLAX, parasternal long-axis; PVC, premature ventricular complex; RBBB, right bundle branch block; RV, right ventricle; RVEDD, right ventricular end-diastolic diameter; RVEDV, right ventricular end-diastolic volume; RVEF, right ventricular ejection fraction; RVOT, right ventricular outflow tract; TAD, terminal activation duration; TFC, task-force criteria; TWI, T-wave inversion; VA, ventricular arrhythmia; VT, ventricular tachycardia.

### Patient outcomes

**Figure 1:**
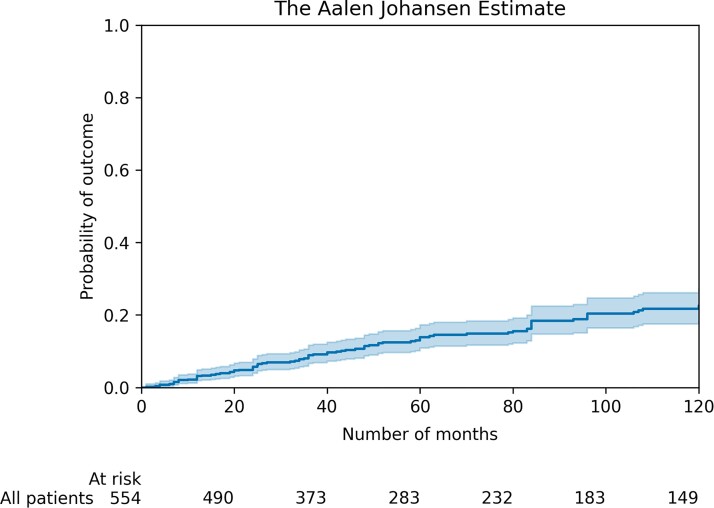
Cumulative incidence curve for the primary outcome. 95% confidence intervals are shown.

During a median follow-up time of 6.0 (3.1,12.5) years, 100 patients (18%) experienced the primary arrhythmic outcome (*Figure 1*) with a corresponding annual event rate of 2.6% (95% CI 1.9–3.3). The primary arrhythmic outcome consisted of spontaneous sustained VT in 37 (6.7%), appropriate ICD intervention in 52 (9.4%), aborted SCD in 2 (0.4%), and SCD in 9 (1.6%). At last follow-up, 17 (3.1%) patients had undergone heart transplantation and 43 (7.8%) had died. Causes of death were SCD in 16 (37%), heart failure in 11 (26%), non-cardiac in 11 (26%), and unknown in 5 (11%).

### Arrhythmogenic right ventricular cardiomyopathy risk score validation

**Figure 2:**
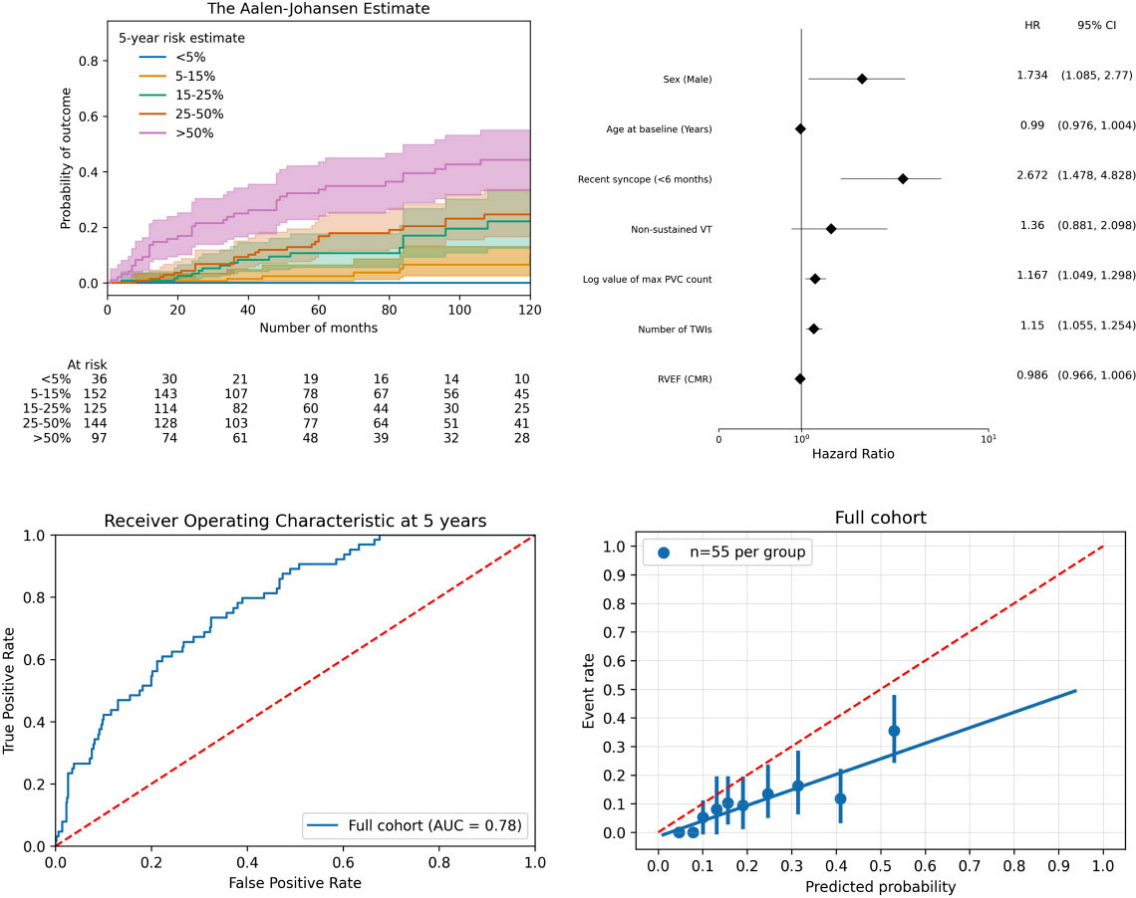
Arrhythmogenic right ventricular cardiomyopathy risk model validation. Cumulative incidence curve for ventricular arrhythmia per strata of risk according to the corrected 2019 ARVC risk model^10,11^ (top left). Forest plot of the impact of each parameter included in the model in a multivariable Fine-Gray regression analysis (top right). Time-dependent area under the curve at 5 years for prediction of the primary arrhythmic outcome (bottom left). Calibration plot showing the agreement between predicted and observed probabilities for the primary outcome (bottom right). Solid line represents results and dotted line represents reference line (bottom left) and perfect calibration (bottom right).

The corrected ARVC risk score was calculated in all 554 individuals. The median calculated corrected 5-year risk score was 17.2% (9.5%–34.3%) (see Supplementary material online, *Figure S3*). Overlapping cumulative incidence of VA in the estimated-risk strata is shown in *Figure 2*. When fitting a multivariable Fine-Gray regression model with the same predictors as the 2019 ARVC risk model, sex (*P* = 0.021), recent syncope (*P* = 0.001), number of TWIs (*P* = 0.001), and log value for PVC count (*P* = 0.004) were found to be significant predictors, whereas age at baseline (*P* = 0.15), NSVT (*P* = 0.16), and RVEF from CMR (*P* = 0.16) were not significant predictors of VA as shown in *Figure 2*. Non-sustained ventricular tachycardia was identified more commonly in patients with ICD than those without (143/234, 61% vs. 120/320, 38%; *P* < 0.001). Uno’s concordance index was 0.75 (95% CI 0.70–0.81). Calibration curve revealed a slope of 0.52 (95% CI 0.37–0.71) and an intercept of −0.01 (95% CI −0.05 to 0.02) suggestive of risk overestimation (*Figure 2*). As expected, the corrected version of the 2019 ARVC risk model resulted in lower risk estimates and less risk overestimation as compared with the original 2019 ARVC risk model (see Supplementary material online, *Figure S6*).

### Impact of genotype and sex on risk stratification

**Figure 3:**
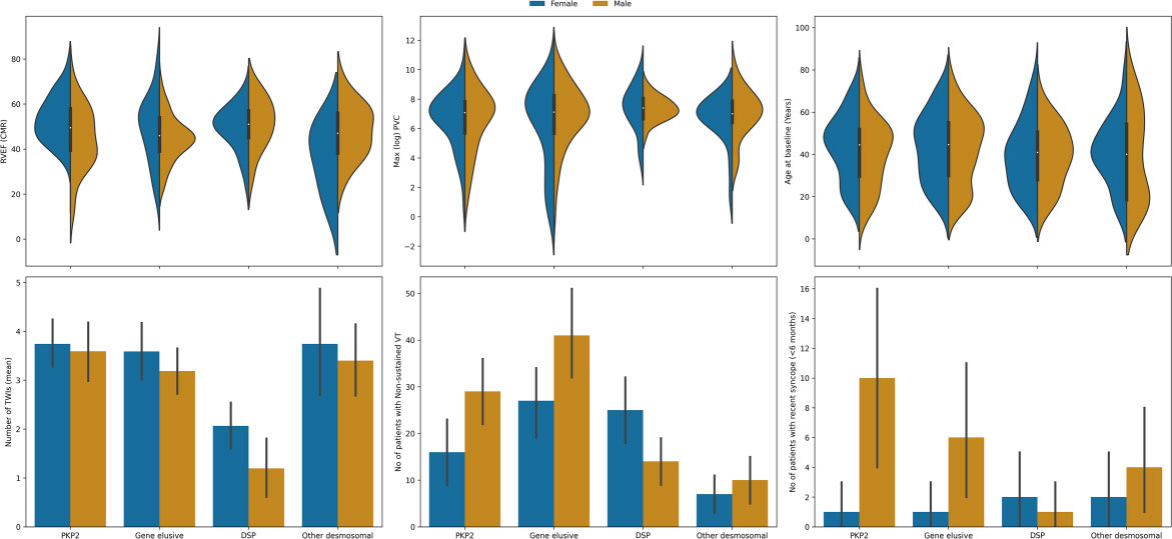
Violin plots (top) and barplots (bottom) of the six key variables for the arrhythmogenic right ventricular cardiomyopathy risk model grouped by genotype group and sex. 95% confidence intervals are shown in barplots.

Genetic analysis was available in 447 (80.7%) of the 554 patients enrolled in the study; a pathogenic or likely pathogenic variant was identified in 290 patients. For the purposes of this analysis, four major gene groups were studied: *PKP2* (*n* = 118, 21.3%), *DSP* (*n* = 79, 14%), other desmosomal gene (*n* = 59, 11%), and gene-elusive patients (*n* = 160, 28.8%). We did not create a subgroup for non-desmosomal gene carriers due to the limited numbers of patients. The clinical phenotypes of each genotype group are summarized in *Figure 3* and *Table 2*. Desmoplakin patients had a higher median RVEF on CMR [51 (45,57) vs. 46 (38,55), *P* = 0.003] and lower number of T-wave inversions in the inferolateral leads [1.0 (0.0–3.0) vs. 3.0 (1.5,5.0), *P* < 0.001] than the rest of the cohort. Patients in the ‘other desmosomal gene’ group had the lowest frequency of non-sustained VT (17/59 vs. 217/495, *P* = 0.027).

**Table 2:** Demographic and clinical characteristics of patients in the main dataset according to the genotype subgroup

	NA	Overall	PKP2	DSP	Gene elusive	Other desmosomal gene	*P*-value
***n* (main dataset)**		**416**	**118**	**79**	**160**	**59**	
Age (years)	0	42.0 (28.0,53.9)	44.6 (29.8,51.9)	41.0 (28.0,50.6)	44.7 (30.0,55.0)	40.0 (18.6,54.4)	0.364
Male sex	0	223 (53.6)	57 (48.3)	31 (39.2)	99 (61.9)	36 (61.0)	0.004
Caucasian ethnicity	1	391 (94.2)	110 (93.2)	74 (94.9)	149 (93.1)	58 (98.3)	0.048
Proband status	0	193 (46.4)	69 (58.5)	48 (60.8)	47 (29.4)	29 (49.2)	<0.001
Symptoms	0	202 (48.6)	46 (39.0)	40 (50.6)	84 (52.5)	32 (54.2)	0.101
Recent syncope (<6 months)	0	27 (6.5)	11 (9.3)	3 (3.8)	7 (4.4)	6 (10.2)	0.172
Structural TFC, major	0	201 (48.3)	52 (44.1)	16 (20.3)	99 (61.9)	34 (57.6)	<0.001
Structural TFC, minor		55 (13.2)	20 (16.9)	8 (10.1)	18 (11.2)	9 (15.3)	
Tissue TFC, major	0	20 (4.8)	4 (3.4)	1 (1.3)	10 (6.2)	5 (8.5)	0.507
Tissue TFC, minor		6 (1.4)	2 (1.7)	1 (1.3)	2 (1.2)	1 (1.7)	
Repolarization TFC, major	0	199 (47.8)	78 (66.1)	13 (16.5)	76 (47.5)	32 (54.2)	<0.001
Repolarization TFC, minor		71 (17.1)	12 (10.2)	23 (29.1)	25 (15.6)	11 (18.6)	
Depolarization TFC, major	0	41 (9.9)	12 (10.2)	1 (1.3)	15 (9.4)	13 (22.0)	0.002
Depolarization TFC, minor		163 (39.2)	42 (35.6)	39 (49.4)	58 (36.2)	24 (40.7)	
Arrhythmia TFC, major	0	49 (11.8)	14 (11.9)	8 (10.1)	21 (13.1)	6 (10.2)	0.062
Arrhythmia TFC, minor		265 (63.7)	68 (57.6)	62 (78.5)	96 (60.0)	39 (66.1)	
Family history TFC, major	0	297 (71.4)	106 (89.8)	71 (89.9)	72 (45.0)	48 (81.4)	<0.001
Family history TFC, minor		24 (5.8)	1 (0.8)	2 (2.5)	16 (10.0)	5 (8.5)	
cRBBB	20	33 (8.3)	5 (4.5)	1 (1.3)	18 (11.8)	9 (16.4)	0.013
LBBB	20	10 (2.6)	3 (2.7)		6 (4.1)	1 (1.9)	0.024
QRS duration	89	100.0 (90.0,110.0)	97.5 (90.0,105.2)	100.0 (90.0,107.2)	104.0 (90.0,116.2)	108.0 (97.5,121.0)	0.004
Terminal activation duration	140	55.0 (40.0,60.0)	52.0 (40.0,58.0)	54.0 (40.0,60.0)	55.0 (40.0,60.0)	56.0 (40.0,64.0)	0.822
Epsilon waves	68	33 (9.5)	8 (8.0)	3 (4.5)	11 (8.7)	11 (20.0)	0.025
Number of TWIs	0	3.0 (1.0,4.0)	4.0 (2.0,5.0)	1.0 (0.0,3.0)	3.0 (1.0,5.0)	4.0 (2.0,5.0)	<0.001
Limb-lead voltages (mV)	140	0.9 (0.6,1.1)	1.0 (0.7,1.1)	0.7 (0.4,0.9)	0.9 (0.7,1.2)	0.8 (0.6,1.2)	<0.001
Precordial-lead voltages (mV)	146	1.5 (1.0,2.0)	1.5 (1.0,2.0)	1.5 (1.1,1.6)	1.4 (1.1,1.9)	1.6 (1.1,2.2)	0.491
PVCs (per 24h)	60	1243.5 (420.2,3121.8)	1174.0 (182.8,2425.8)	1604.0 (754.0,3121.0)	1188.0 (196.0,3748.0)	1100.0 (566.0,2500.0)	0.111
Non-sustained VT	0	169 (40.6)	45 (38.1)	39 (49.4)	68 (42.5)	17 (28.8)	0.092
RV dilatation (ECHO)—none	29	174 (45.0)	55 (51.4)	51 (68.0)	55 (36.9)	13 (23.2)	<0.001
RV dilatation (ECHO)—mild		92 (23.8)	15 (14.0)	17 (22.7)	43 (28.9)	17 (30.4)	
RV dilatation (ECHO)—moderate		75 (19.4)	26 (24.3)	6 (8.0)	30 (20.1)	13 (23.2)	
RV dilatation (ECHO)—severe		46 (11.9)	11 (10.3)	1 (1.3)	21 (14.1)	13 (23.2)	
RVOT-PLAX diameter (ECHO)	156	32.0 (28.8,37.0)	32.0 (28.0,37.0)	30.0 (27.0,32.8)	33.0 (29.1,37.8)	34.5 (31.2,37.8)	0.001
LVEDD (ECHO)	114	50.0 (47.0,55.0)	48.0 (45.0,52.5)	53.0 (48.0,57.0)	51.0 (47.0,55.0)	48.0 (47.0,51.0)	<0.001
LVEF (ECHO)	19	58.0 (50.0,65.0)	62.0 (55.8,66.2)	52.0 (42.8,58.6)	56.5 (50.0,64.2)	60.0 (52.2,65.0)	<0.001
ICD implantation	0	209 (50.2)	59 (50.0)	54 (68.4)	68 (42.5)	28 (47.5)	0.002
Follow-up duration (years)	0	6.0 (3.0,11.0)	5.9 (2.9,10.9)	5.0 (2.8,9.9)	6.0 (3.4,12.9)	7.0 (3.0,12.5)	0.485
VA		67 (16.1)	27 (22.9)	11 (13.9)	19 (11.9)	10 (16.9)	
Heart transplantation	0	7 (1.7)		2 (2.5)	5 (3.1)		0.146
Mortality	0	21 (5.0)	5 (4.2)	4 (5.1)	2 (1.2)	10 (16.9)	<0.001
VT ablation	0	17 (4.1)	3 (2.5)	2 (2.5)	7 (4.4)	5 (8.5)	0.247
***n* (CMR dataset)**		**288**	**116**	**80**	**62**	**30**	
RVEDV (CMR)	40	182.0 (144.8,219.2)	182.0 (147.8,213.2)	170.0 (124.2,184.0)	185.5 (145.8,229.8)	197.0 (165.0,231.8)	0.004
RVEF (CMR)	26	48.0 (40.0,56.0)	49.5 (39.5,58.0)	51.0 (45.0,57.0)	46.0 (39.0,54.0)	47.0 (38.2,56.0)	0.066
RV LGE	8	98 (35.0)	26 (33.8)	18 (29.0)	39 (33.9)	15 (57.7)	0.073
LVEDV (CMR)	23	156.0 (129.0,184.0)	145.0 (128.5,168.0)	180.0 (139.5,202.5)	156.0 (132.0,190.0)	149.0 (124.5,169.5)	0.008
LVEF (CMR)	12	58.0 (49.8,63.2)	63.0 (58.0,67.0)	53.0 (43.0,60.0)	55.0 (49.0,61.8)	60.0 (53.0,66.0)	<0.001
LV LGE	8	153 (54.6)	23 (29.9)	60 (96.8)	55 (47.8)	15 (57.7)	<0.001

Categorical variables are reported as *n* (%). Continuous variables are reported as mean ± SD or median (25th, 75th percentiles) according to the distribution normality. CMR, cardiac magnetic resonance; cRBBB, complete right bundle branch block; ECHO, echocardiography; ICD, intracardiac cardioverter defibrillator; LBBB, left bundle branch block; LGE, late gadolinium enhancement; LV, left ventricle; LVEDD, left ventricular end-diastolic diameter; LVEDV, left ventricular end-diastolic volume; LVEF, left ventricular ejection fraction; NA, not available; PLAX, parasternal long-axis; PVC, premature ventricular complex; RBBB, right bundle branch block; RV, right ventricle; RVEDD, right ventricular end-diastolic diameter; RVEDV, right ventricular end-diastolic volume; RVEF, right ventricular ejection fraction; RVOT, right ventricular outflow tract; TAD, terminal activation duration; TFC, task-force criteria; TWI, T-wave inversion; VA, ventricular arrhythmia; VT, ventricular tachycardia.

**Figure 4:**
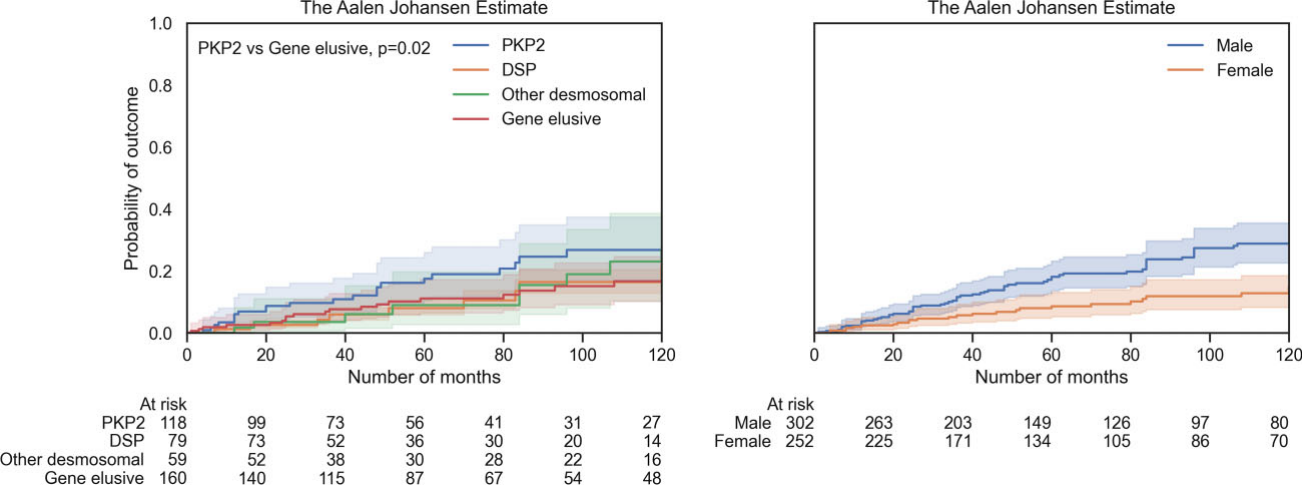
Cumulative incidence curves for ventricular arrhythmia stratified by main genotype groups (left) and sex (right). 95% confidence intervals are shown.

Cumulative incidence for VA per gene group is shown in *Figure 4*. Overall, there was no significant difference (Gray’s test, *P* = 0.37), but in pairwise comparisons, the gene-elusive group had significantly lower cumulative VA incidence compared with the *PKP2* group (Gray’s test, *P* = 0.02). Annual VA rate was 3.6% (95% CI 2.2–5.0) in *PKP2* patients, 2.3% (95% CI 0.9–3.6) in *DSP* patients, 2.1% (95% CI 0.8–3.5) in patients with variants in either *DSG2*, *DSC2*, or *JUP* and 1.5% (95% CI 0.8–2.1) in gene-elusive patients. Cumulative incidence of VA analysis of the cohort stratified by individual genes did reveal an overall difference (see Supplementary material online, *Figure S4*, Gray’s test, *P* = 0.03). Male sex was associated with a higher cumulative incidence of VA (*Figure 4*, Gray’s test, *P* = 0.001).

**Figure 5:**
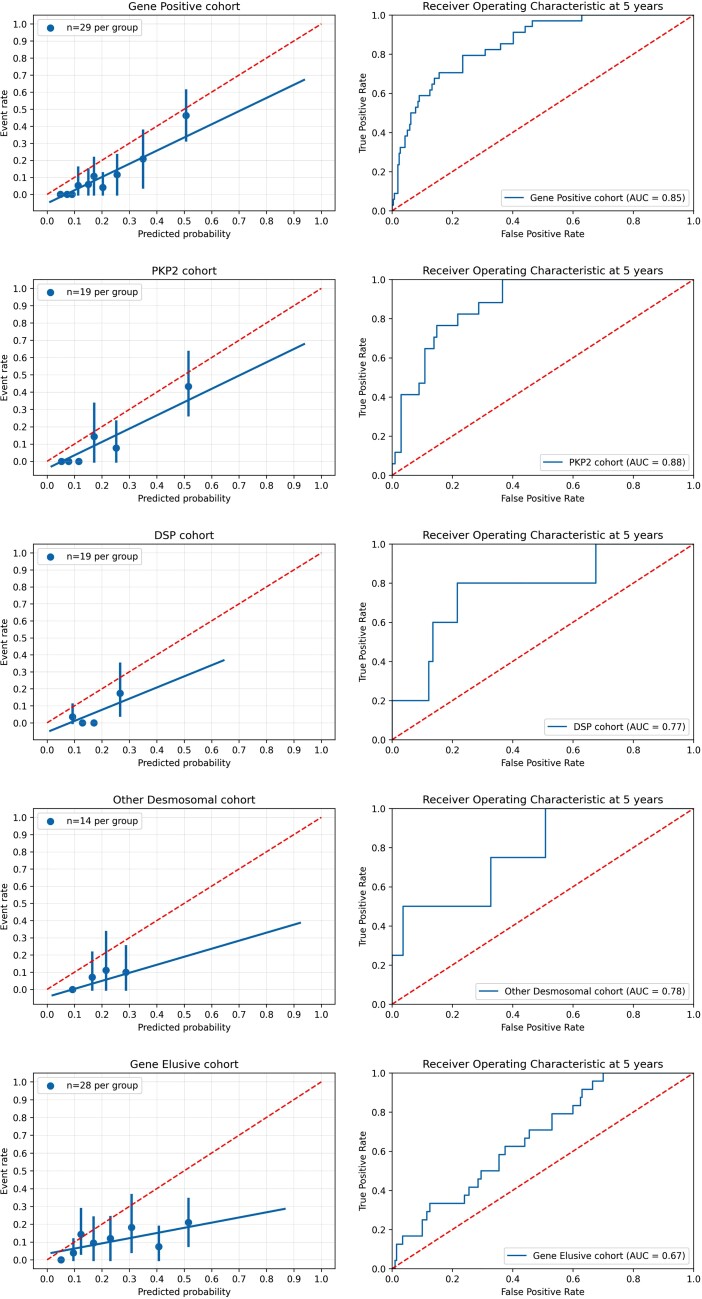
Corrected arrhythmogenic right ventricular cardiomyopathy risk model validation per major genotype group. Calibration plot showing the agreement between predicted and observed probabilities for the primary outcome (left). Time-dependent area under the curve for prediction of the primary arrhythmic outcome (right). Solid line represents results and dotted line represents perfect calibration (left) and reference line (right).

Validation of the corrected 2019 ARVC risk score in the various gene groups revealed significant differences in the performance of the model. As shown in *Figure 5*, the best fit was demonstrated within the gene-positive group (Uno’s concordance index 0.82, 95% CI 0.76–0.88; calibration curve slope 0.78, 95% CI 0.53–1.06; calibration curve intercept −0.05, 95% CI −0.10 to −0.01) and the worst fit was found in the gene-elusive group (Uno’s concordance index 0.65, 95% CI 0.57–0.74; calibration curve slope 0.27, 95% CI 0.06–0.55; calibration curve intercept 0.04, 95% CI −0.02 to –0.09). The best performance was seen for *PKP2* (Uno’s concordance index 0.83, 95% CI 0.75–0.91; calibration curve slope 0.73, 95% CI 0.44–1.14; calibration curve intercept −0.03, 95% CI −0.10 to 0.02) followed by *DSP* (Uno’s concordance index 0.80, 95% CI 0.53–0.96; calibration curve slope 0.69, 95% CI 0.00–1.37; calibration curve intercept −0.06, 95% CI −0.14 to 0.05) and other desmosomal variants (Uno’s concordance index 0.73, 95% CI 0.48–1.00; calibration curve slope 0.28, 95% CI −0.06 to 0.77; calibration curve intercept 0.00, 95% CI −0.09 to 0.08). Sensitivity analyses showed that these findings were recapitulated in the complete-case and CMR datasets but did not reach statistical significance due to smaller sample size (see Supplementary material online, *Table S4*).

**Figure 6:**
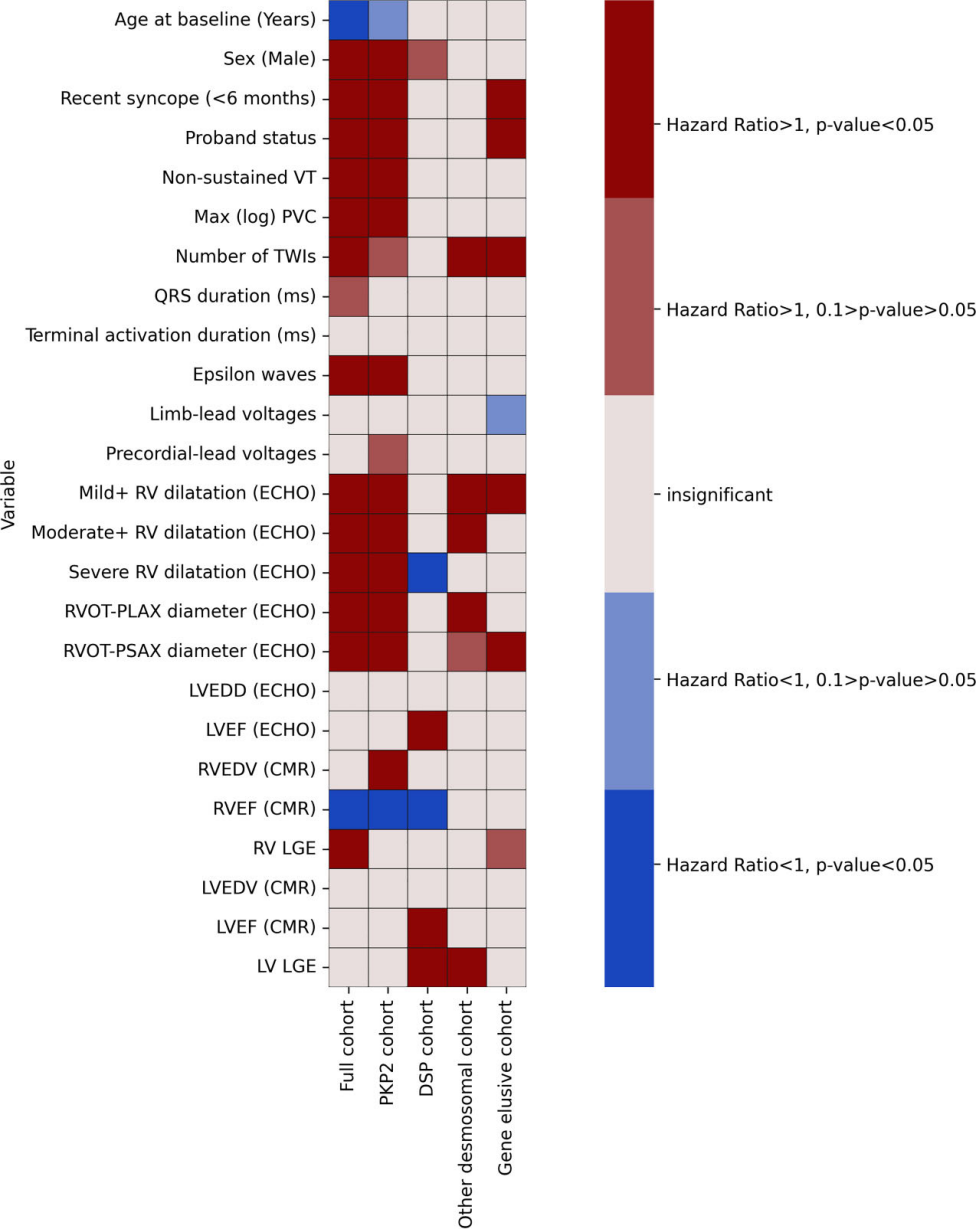
Heatmap of univariate predictors in the full cohort and major gene groups displaying the hazard ratios and relevant statistical significance derived from univariate analyses.

The variable performance of the corrected 2019 ARVC risk model in different genotypes led us to hypothesize that there may be differences in the performance of individual parameters as predictors of risk among the different gene groups. Due to the limited number of events in each gene group we opted not to perform multivariable analyses, but instead conducted multiple univariate analyses of all the available clinical characteristics in each gene group. Significant differences were present among the gene groups (*Figure 6*). The *PKP2* group had the most significant predictors and the DSP group the least among the clinical variables that were studied (*Figure 6*). Sex was mainly a *PKP2* group-related risk predictor (*Figure 6*, see Supplementary material online, *Figure S5*). Left ventricular parameters reached statistical significance almost only within the DSP group. Similarly, the utility of right ventricular parameters was limited in the non-PKP2 groups (*Figure 6*).

## Discussion

In this multicentre cohort of patients with ARVC, we show that the corrected 2019 ARVC risk score^10,11^ has a reasonable discriminative ability for VA, but it suffers from risk overestimation for VA. The analysis by genotype shows that the corrected 2019 model performs best in patients with pathogenic or likely pathogenic gene variants, especially *PKP2*, but is more limited in patients with an elusive genetic status. Analyses of individual potential risk predictors among the gene groups revealed significant variability reflecting differences in clinical phenotypes (*Structured Graphical Abstract*).

### Risk stratification in arrhythmogenic cardiomyopathy

Although the notion that all patients presenting with tolerated sustained VA have a high risk of SCD has been challenged, it remains standard practice to offer them ICDs.^29^ There has been less certainty about patient selection for primary prevention ICDs.

A large number of clinical predictors have been suggested as risk markers in ARVC, but supporting evidence is inadequately validated and often based on small heterogeneous cohorts.^30,31^ The original 2019 ARVC risk model was the first systematic attempt to develop a validated clinical tool that provides individual risk estimates for sustained VA in patients with definite ARVC and no prior sustained VA.^10^ A correction of the baseline survival probability has recently been published.^11^ External validation of the corrected 2019 ARVC risk score in our cohort revealed a generally good accuracy, but we observed a much lower annual event rate and a significant overestimation of risk compared with the original paper.^10^ Risk overestimation was even more pronounced in the original 2019 ARVC risk model. Other, smaller studies have reported a good performance of the model.^31–33^ Similarly, when all the risk predictors used in the 2019 ARVC risk score were fitted to a Fine-Gray regression model, only sex, syncope, the number of TWIs, and log PVC count remained independent risk factors, whereas NSVT, age at diagnosis, and RVEF on CMR were non-significant.

To provide close comparison, we sought to replicate the characteristics of the 2019 development cohort. Nevertheless, there may be some important biases in patient selection. For example, the participating centres in our study were predominantly cardiomyopathy units compared with the electrophysiology focused units that participated in the 2019 study, which could predispose to a more arrhythmia-prone population.^10^ This can potentially explain the lower annual incidence rate of VA and thus the risk overestimation that we have observed compared with the 2019 model development study.^10^ In addition, the genotype composition between the two studies differs; for example, the prevalence of *PKP2* variants in our cohort is 21.7% compared with 48.9% in the 2019 study^10^. The fact that *PKP2* variant carriers exhibited a higher cumulative incidence of VA than gene-elusive patients might also affect the corrected 2019 ARVC risk model performance in our cohort.

### Utilizing genotype information for precision arrhythmic risk stratification

While ARVC has been uniformly defined using the 2010 TFC diagnostic criteria, it is recognized that specific genotypes associate with different phenotypic features.^2,34^ For example, prognostic markers such as TWI and early age of disease onset are more commonly seen in patients with ARVC caused by desmosomal gene variants.^35^ Although some rare genotypes associated with particularly arrhythmic profiles exist,^36,37^ there has been limited evidence to link specific genes to arrhythmic outcome prediction.^4–6,38^ In a recent study by the Nordic ARVC Registry, *PKP2* mutation carriers showed decreased arrhythmia-free survival.^39^ This is consistent with our findings suggesting a higher cumulative incidence for VA in *PKP2* vs. gene-elusive patients (*Figure 4*).

In our cohort, we observed that gene-elusive patients have the lowest incidence of the primary outcome, whereas the incidence was similar between the major gene groups. Application of the corrected 2019 ARVC risk model in the specific gene groups revealed good performance within the gene-positive patients and particularly the *PKP2* group with least overestimation of risk compared with the total cohort (mostly identified in the lower risk strata) in comparison with the gene-elusive group where the model had modest performance with significant risk overestimation across all risk strata. Model performance was intermediate for the *DSP* and other desmosomal groups but did not differ significantly with the other subgroups, possibly due to smaller patient numbers. The corrected 2019 ARVC risk model should be used with caution in non-*PKP2* patients.

Due to restricted subgroup sizes, we were unable to perform multivariable analyses to study individual risk markers in different genotype clusters, but in a univariable analysis we did observe gene-specific differences in the association of some of the variables used by the 2019 ARVC risk model with VA. For example, significant risk was conferred by right ventricular parameters mainly in the *PKP2* group. Interestingly, sex did not reach statistical significance in any of the non-*PKP2* groups (*Figure 6*, see Supplementary material online, *Figure S5*). To our knowledge, this is the first demonstration of this phenomenon and as such generates hypotheses for future studies.

The main implication of our findings is that the incorporation of genotype is vital in future iterations of risk models in ARVC. Variables such as variant type and location and the presence of multiple variants have all been shown to affect phenotype and would likely demonstrate unique risk profiles.^4,5,39^ Ethnic differences might also add to this complexity. Similarly, forms of ARVC that affect predominantly the left ventricle may require bespoke risk prediction models.^40^

### Limitations

The limited size of the cohort did not allow for gene-specific analyses for all genes harbouring causative variants. Even in the groups with significant differences in model performance, a limited number of events was observed and the results should be interpreted cautiously. The lack of CMR at the baseline timepoint in a significant part of the cohort challenged the application of the corrected 2019 ARVC risk score, which was addressed using data imputation. Our study population is overwhelmingly Caucasian, and therefore extrapolation to other ethnic backgrounds should be done with caution. Information on ICD programming was not analysed but all centres are expert in ICD implantation and management and offered contemporary programming strategies to all patients. That said, considering the multicentre and retrospective nature of this study, patients are likely to have been exposed to varying ICD programming practices that may have influenced the frequency of the primary arrhythmic outcome. Non-sustained ventricular tachycardia has been defined as an ‘any exam diagnosis’ and therefore patients with an ICD have a higher likelihood of NSVT detection than those without.

## Conclusion

The corrected 2019 ARVC risk score has a reasonable discriminative ability but suffers from risk overestimation. It performs best among gene-positive patients and especially in the *PKP2* subgroup, but its utility is limited in gene-elusive patients. The predictive power of individual risk markers also varies by genotype. Future iterations of risk models in ARVC should incorporate genotype data.

## Supplementary material

Supplementary material will be available at *European Heart Journal online*.

## Supplementary Material

ehac235_Supplementary_Data
