# Supplementary material for: Importance of genotype for risk stratification in arrhythmogenic right ventricular cardiomyopathy using the 2019 ARVC risk calculator
Source: Eur Heart J. 2022 Jun 29;43(32):3053–67. doi: 10.1093/eurheartj/ehac235 (PMC9392652; doi:10.1093/eurheartj/ehac235)
Supplement: ehac235_Supplementary_Data [file ehac235_supplementary_data.zip › Revised_Supplement_(Highlighted).docx]

**Supplemental Material**

# Supplementary Methods

## Missing data

Missing data were assessed in all the variables required in the validation of the ARVC risk score, including the phenotypic variables used to calculate the score and the gene elusive status. Missing values were identified in the “PVC” (n=77, 14%), “RVEF” (n=263, 48%), “number of T-wave inversions in the anterior and inferior leads” (n=12, 2%) and “gene positive status” (n=104, 19%) variables. Data missingness was poorly corelated between these variables (Supplementary Figure 1).

## Data imputation methodology

The CMR dataset was used as a supplementary dataset which reflects the same cohort at a secondary timepoint where the missingness of RVEF from CMR is minimal. This has been used in the data imputation process and in the sensitivity analyses.

Data derived from the CMR dataset (n=359) was used to estimate the correlation of RVEF with fractional area change (FAC), with right ventricular outflow tract diameter in the parasternal long axis view (RVOT PLAX) and visual assessment of right ventricular size on ECHO. The following imputation steps were performed: 1) Manual imputation of “RVEF” based on the value from the first CMR during follow-up as derived in the CMR dataset. 2) Manual imputation of “RVEF” based on a conversion factor from FAC, if a FAC measurement was available. 3) Manual imputation of “RVEF” based on a conversion factor from RVOT PLAX diameter, if available. 4) Manual imputation of “RVEF” based on a conversion factor from visual assessment of RV dilatation from ECHO. 5) Imputation using the multiple imputation with chained equations method (MICE) performed with all available other clinical variables with less than 10% data missingness (Supplementary Figure 1). Furthermore, gene elusive status was missing in 104 cases (18.8%) and it was included in the data imputation procedure in order to minimize bias. These 5 steps were used to create 5 different imputed datasets. Model 1 utilized steps 1,2,5, model 2 utilized steps 1,2,3,5, model 3 utilized steps 1,2,3,4,5, model 4 utilized steps 1,5 and model 5 utilized step 5 only (no manual imputation). Kernel density estimation (KDE) plots were used to compare the imputed variables between the different methods as well as the resulting estimated corrected 5-year ARVC risk score (Supplementary Figure 2).

## Sensitivity analyses

Sensitivity analyses were performed between the final (imputed dataset), CMR dataset and complete-case dataset. Results of the key group comparators are shown in supplementary Table S4.

## Original 2019 5-year ARVC risk score

During the review of this paper a corrected version of the 2019 risk model was published [add reference]. The correction was to the baseline survival probability at five years which was 0.801 in the original version. According to the original score the median calculated original 5-year risk score was 21.3% [11.9%-41.3%]. The calibration plots based on the original 5-year risk score, with the respective calibration slope and intercept are presented in Supplementary Figure 6.

# Supplementary Tables

**Supplementary Table S1:** Description of centres included in the study with respective number of patients included.

| Cardiomyopathy Centre | n |
| --- | --- |
| University College London AC Registry, London, UK | 178 |
| University of Trieste, Trieste, Italy | 82 |
| University of Padua, Padua, Italy | 51 |
| University Hospital Virgen de la Victoria, Malaga, Spain | 45 |
| University Hospital Virgen de Arrixaca, Murcia, Spain | 33 |
| Hospital Universitario y Politécnico La Fe de Valencia, Valencia Spain | 28 |
| Onassis Cardiothoracic centre, Athens, Greece | 22 |
| Nikos Protonotarios Medical centre, Naxos, Greece | 22 |
| Sant'Orsola Hospital, Bologna, Italy | 21 |
| Hospital Cochin, Paris, France | 16 |
| Hospital Universitario Puerta de Hierro, Madrid, Spain | 11 |
| Odense University Hospital, Odense, Denmark | 10 |
| Cyprus Institute of Cardiomyopathies and inherited cardiovascular diseases | 9 |
| University Hospital complex Coruna, A Coruna, Spain | 8 |
| Son Llatzer University Hospital, Palma de Mallorca, Spain | 7 |
| Kochi Medical School, Nankoku, Japan | 6 |
| University Hospital Vall d'Hebron, Barcelona, Spain | 5 |

**Supplementary Table S2:** Data dictionary and definitions used at the electronic data capture tool.

| **Field Label** | **Choices, Calculations, OR Slider Labels** | **Field Note** |
| --- | --- | --- |
| Record ID |  | Pseudonymized at a local level |
| Centre | 1, London \| 2, Padua \| 3, Athens \| 4, Naxos \| 5, Cyprus \| 6, Odense \| 7, Paris \| 8, Bologna \| 9, A Coruna \| 10, Valencia \| 11, Mallorca \| 12, Granada \| 13, Malaga \| 14, Barcelona \| 15, Madrid \| 16, Murcia \| 17, Trieste \| 18, Kochi |  |
| Sex | 1, Male \| 2, Female |  |
| Ethnicity | 1, Caucasian \| 2, African \| 3, Asian |  |
| Pedigree status | 1, Proband \| 2, Family member |  |
| Genetic testing | 1, Not performed \| 2, Performed - No findings \| 3, Performed - Findings present | Details of genetic variants to be entered at the next instrument |
| Comments on genetic testing |  |  |
| Gene | (list of 76 genes provided) |  |
| Coding change |  |  |
| Protein change |  |  |
| Zygosity | 1, Heterozygous \| 2, Homozygous-recessive \| 3, Homozygous-dominant |  |
| Classification | 1, Pathogenic \| 2, Likely pathogenic \| 3, Uncertain significance \| 4, Likely benign \| 5, Benign | According to ACMG |
| Comments |  |  |
| Age at baseline evaluation (Months) |  | If patient did not fulfill 2010 TFC at baseline, use age of dx |
| Symptoms at baseline |  |  |
| Cardiac syncope |  | Transient loss of consciousness and postural tone with spontaneous recovery with arrhythmic mechanism likely at diagnosis. This thus excludes syncope of vaso-vagal etiology. |
| Age at cardiac syncope (Months) |  |  |
| Was ECG performed at baseline assessment? |  | =/- 1 year from time of baseline assessment |
| Rhythm | 1, Sinus rhythm \| 2, Atrial Fibrillation \| 3, Atrial Flutter \| 4, Atrial Tachycardia \| 5, Supraventricular Tachycardia \| 6, Junctional rhythm \| 7, Junctional Tachycardia \| 8, Ventricular Tachycardia \| 9, Ventricular Fibrillation \| 10, Accelerated idioventricular rhythm |  |
| Right Bundle branch block | 1, None \| 2, iRBBB \| 3, RBBB |  |
| Left Bundle branch block | 1, None \| 2, LAFB \| 3, LPFB \| 4, iLBBB \| 5, LBBB \| 6, IVCD |  |
| Maximum QRS duration (ms) |  |  |
| Terminal Activation Duration (Max in V1-V3) (ms) |  |  |
| Epsilon waves |  |  |
| T-waves V1 | 1, Upright \| 2, Flat \| 3, Biphasic \| 4, Inverted | T-waves are considered inverted or upright if amplitude ≥ 1 mV (1 mm), otherwise flat unless biphasic |
| T-waves V2 | 1, Upright \| 2, Flat \| 3, Biphasic \| 4, Inverted | T-waves are considered inverted or upright if amplitude ≥ 1 mV (1 mm), otherwise flat unless biphasic |
| T-waves V3 | 1, Upright \| 2, Flat \| 3, Biphasic \| 4, Inverted | T-waves are considered inverted or upright if amplitude ≥ 1 mV (1 mm), otherwise flat unless biphasic |
| T-waves V4 | 1, Upright \| 2, Flat \| 3, Biphasic \| 4, Inverted | T-waves are considered inverted or upright if amplitude ≥ 1 mV (1 mm), otherwise flat unless biphasic |
| T-waves V5 | 1, Upright \| 2, Flat \| 3, Biphasic \| 4, Inverted | T-waves are considered inverted or upright if amplitude ≥ 1 mV (1 mm), otherwise flat unless biphasic |
| T-waves V6 | 1, Upright \| 2, Flat \| 3, Biphasic \| 4, Inverted | T-waves are considered inverted or upright if amplitude ≥ 1 mV (1 mm), otherwise flat unless biphasic |
| T-waves I | 1, Upright \| 2, Flat \| 3, Biphasic \| 4, Inverted | T-waves are considered inverted or upright if amplitude ≥ 1 mV (1 mm), otherwise flat unless biphasic |
| T-waves II | 1, Upright \| 2, Flat \| 3, Biphasic \| 4, Inverted | T-waves are considered inverted or upright if amplitude ≥ 1 mV (1 mm), otherwise flat unless biphasic |
| T-waves III | 1, Upright \| 2, Flat \| 3, Biphasic \| 4, Inverted | T-waves are considered inverted or upright if amplitude ≥ 1 mV (1 mm), otherwise flat unless biphasic |
| T-waves aVR | 1, Upright \| 2, Flat \| 3, Biphasic \| 4, Inverted | T-waves are considered inverted or upright if amplitude ≥ 1 mV (1 mm), otherwise flat unless biphasic |
| T-waves aVL | 1, Upright \| 2, Flat \| 3, Biphasic \| 4, Inverted | T-waves are considered inverted or upright if amplitude ≥ 1 mV (1 mm), otherwise flat unless biphasic |
| T-waves aVF | 1, Upright \| 2, Flat \| 3, Biphasic \| 4, Inverted | T-waves are considered inverted or upright if amplitude ≥ 1 mV (1 mm), otherwise flat unless biphasic |
| Maximum limb lead voltages (mV) |  | QRS amplitude (Difference between lowest and highest point of QRS) |
| Maximum precordial lead voltages (mV) |  | QRS amplitude (Difference between lowest and highest point of QRS) |
| ECG comments |  |  |
| Was Holter performed at baseline |  | +/- 1 year from the time of baseline assessment |
| Maximum PVC count (per 24h) |  |  |
| NSVT? (Any exam diagnosis) |  | 3 or more consecutive ventricular beats at a rate of >100 beats per minute with duration of less than 30 seconds and without hemodynamic compromise |
| Holter comments |  |  |
| ECHO available at baseline |  | =/- 1 year since baseline assessment |
| ECHO dilation RV | 1, Normal \| 2, Mild dilatation \| 3, Moderate dilatation \| 4, Severe dilatation | Visual assessment |
| ECHO RVOT PLAX diameter (mm) |  |  |
| ECHO RVOT PSAX diameter (mm) |  |  |
| ECHO RV FAC (%) |  |  |
| ECHO LVEDD (mm) |  |  |
| ECHO LVEF (%) |  |  |
| ECHO comments |  |  |
| Was CMR performed at baseline? |  | =/- 1 year from baseline assessment |
| BSA |  |  |
| CMR RVEDV (ml) |  |  |
| CMR RVEF(%) |  |  |
| CMR RV LGE |  |  |
| CMR LVEDV (ml) |  |  |
| CMR LVEF(%) |  |  |
| CMR LV LGE |  |  |
| CMR comments |  |  |
| Global or regional dysfunction and structural alterations | 0, none \| 1, Minor \| 2, Major |  |
| Tissue characterization of wall | 0, none \| 1, Minor \| 2, Major |  |
| Repolarization abnormalities | 0, none \| 1, Minor \| 2, Major |  |
| Depolarization abnormalities Major | 0, none \| 1, Minor \| 2, Major |  |
| Arrhythmias | 0, none \| 1, Minor \| 2, Major |  |
| Family history | 0, none \| 1, Minor \| 2, Major |  |
| ICD implanted at any time? |  |  |
| Age at first ICD implantation (months) |  |  |
| Cycle length of the Monitor zone at implant (ms) |  |  |
| Cycle length of the lowest therapy zone at implant (ms) |  |  |
| Cycle length of the monitor zone at first LTVA or last programing available at follow-up (ms) | |  |
| Cycle length of the lowest therapy zone at first LTVA or last programing available at follow-up (ms) | |  |
| Comments about ICD |  |  |
| Anti-arrhythmic medication taken at baseline | 0, none \| 1, Amiodarone \| 2, Sotalol \| 3, Class IC( Propafenone or Flecainide) \| 4, Dofetilide \| 5, Mexiletine \| 6, other |  |
| Anti-arrhythmic medication taken at the time of event or censoring | 0, none \| 1, Amiodarone \| 2, Sotalol \| 3, Class IC( Propafenone or Flecainide) \| 4, Dofetilide \| 5, Mexiletine \| 6, other |  |
| Beta blockers taken at baseline |  | excluding sotalol |
| Beta blockers taken at the time of event or censoring |  | excluding sotalol |
| Comments about medications |  |  |
| Life threatening ventricular arrhythmia (LTVA)? | 0, None \| 1, Spontaneous sustained VT \| 2, ICD intervention \| 3, Aborted sudden cardiac arrest (SCA) \| 4, Sudden cardiac death (SCD) |  |
| Cycle length of ventricular arrhythmia coded as the primary outcome (ms) | |  |
| Age at first LTVA (months) |  |  |
| Cardiac transplant during follow-up |  |  |
| Age at transplant (months) |  |  |
| Death during follow-up |  |  |
| Age of death (months) |  |  |
| Cause of death category | 1, 1=SCD, \| 2, 2=heart failure, \| 3, 3=arrhythmic and heart failure (eg. heart failure largely caused by arrhythmias, \| 4, 4=non-cardiac death |  |
| Cause of death (text) |  |  |
| VT ablation (endocardial or epicardial) performed at any time before last coded event? | |  |
| Age of first VT ablation (months) |  |  |
| Age at last follow-up (months) |  |  |
| Additional notes about outcomes |  |  |

**Supplementary Table S3:** Description of all identified genetic variants that were classified as “pathogenic” or “likely pathogenic” according to the American College of Medical Genetics and Genomics guidelines.

| ID | Gene | Zygosity | Pathogenicity | Nucleotide change | Protein |
| --- | --- | --- | --- | --- | --- |
| 68 | DES | Heterozygous | Likely Pathogenic | c.343C>A | p.Leu115Ile |
| 15 | DES | Heterozygous | Likely Pathogenic | c.343C>A | p.Leu115Ile |
| 16 | DES | Heterozygous | Likely Pathogenic | c.343C>A | p.Leu115Ile |
| 17 | DES | Heterozygous | Likely Pathogenic | c.343C>A | p.Leu115Ile |
| 18 | DES | Heterozygous | Likely Pathogenic | c.343C>A | p.Leu115Ile |
| 255 | DSC2 | Heterozygous | Pathogenic | c.1841delG | p.Ser614fsX625 |
| 152 | DSC2 | Heterozygous | Pathogenic | c.133delG | p.Pro45fsX54 |
| 151 | DSC2 | Heterozygous | Pathogenic | c.133delG | p.Pro45fsX54 |
| 91 | DSC2 | Heterozygous | Pathogenic | c.133delG | p.Pro45fsX54 |
| 148 | DSC2 | Heterozygous | Pathogenic | c.133delG | p.Pro45fsX54 |
| 118 | DSC2 | Heterozygous | Pathogenic | c.1112_1113delTG | p.Gly371fsX378 |
| 120 | DSC2 | Heterozygous | Pathogenic | c.1112_1113delTG | p.Gly371fsX378 |
| 90 | DSC2 | Heterozygous | Pathogenic | c.133delG | p.Pro45fsX54 |
| 89 | DSC2 | Heterozygous | Pathogenic | c.133delG | p.Pro45fsX54 |
| 84 | DSC2 | Heterozygous | Likely Pathogenic | c.536A>G | p.Asp179Gly |
| 251 | DSC2 | Heterozygous | Likely Pathogenic | c.1430delC | p.Thr477fsX480 |
| 163 | DSC2 | Heterozygous | Pathogenic | c.1123C>T | p.Arg375X |
| 132 | DSG2 | Heterozygous | Pathogenic | c.1003A>G | p.Thr335Ala |
| 133 | DSG2 | Homozygous Recessive | Pathogenic | c.1003A>G | p.Thr335Ala |
| 134 | DSG2 | Homozygous Recessive | Pathogenic | c.1003A>G | p.Thr335Ala |
| 205 | DSG2 | Heterozygous | Pathogenic | c.685A>T | p.Arg229X |
| 71 | DSG2 | Heterozygous | Pathogenic | c.2875_2876delCA | p.Gln959GlufsX22 |
| 283 | DSG2 | Heterozygous | Pathogenic | c.1003A>G | p.Thr335Ala |
| 217 | DSG2 | Heterozygous | Likely Pathogenic | c.1003A>G | p.Thr335Ala |
| 278 | DSG2 | Heterozygous | Pathogenic | c.3059_3062delAGAG | p.Glu1020AlafsX18 |
| 244 | DSG2 | Heterozygous | Pathogenic | c.1773_1774delTG | p.Cys591X |
| 131 | DSG2 | Heterozygous | Pathogenic | c.1003A>G | p.Thr335Ala |
| 7 | DSG2 | Heterozygous | Pathogenic | c.3G>C | p.0? |
| 246 | DSG2 | Heterozygous | Pathogenic | c.3G>C | p.0? |
| 59 | DSG2 | Heterozygous | Pathogenic | c.3G>C | p.0? |
| 238 | DSG2 | Heterozygous | Pathogenic | c.146G>A | p.Arg49His |
| 73 | DSG2 | Heterozygous | Likely Pathogenic | c.2032delG | p.Gly679AlafsX3 |
| 78 | DSG2 | Heterozygous | Pathogenic | c.797A>G | p.Asn266Ser |
| 81 | DSG2 | Heterozygous | Likely Pathogenic | c.1038_1040delGAA | p.Lys346del |
| 82 | DSG2 | Heterozygous | Pathogenic | c.797A>G | p.Asn266Ser |
| 83 | DSG2 | Heterozygous | Likely Pathogenic | c.2323delT | p.Tyr775IlefsX33 |
| 33 | DSG2 | Heterozygous | Pathogenic | c.829_840del12 | p.Leu277_Met280del |
| 33 | DSG2 | Heterozygous | Pathogenic | c.1003A>G | p.Thr335Ala |
| 161 | DSG2 | Heterozygous | Likely Pathogenic | c.1652-2_1652-1insA |  |
| 144 | DSG2 | Heterozygous | Pathogenic | c.889G>A | p.Asp297Asn |
| 239 | DSG2 | Heterozygous | Likely Pathogenic | c.484delG | p.Asp162MetfsX10 |
| 214 | DSG2 | Heterozygous | Pathogenic | c.685A>T | p.Arg229X |
| 203 | DSG2 | Heterozygous | Pathogenic | c.1310G>A | p.Trp437X |
| 181 | DSG2 | Heterozygous | Likely Pathogenic | c.1297G>A | p.Asp433Asn |
| 202 | DSG2 | Heterozygous | Pathogenic | c.685A>T | p.Arg229X |
| 254 | DSG2 | Heterozygous | Pathogenic | c.1003A>G | p.Thr335Ala |
| 254 | DSG2 | Heterozygous | Pathogenic | c.1038_1040delGAA | p.Lys346del |
| 193 | DSG2 | Heterozygous | Pathogenic | c.685A>T | p.Arg229X |
| 207 | DSG2 | Heterozygous | Likely Pathogenic | c.1003A>G | p.Thr335Ala |
| 220 | DSG2 | Heterozygous | Pathogenic | c.685A>T | p.Arg229X |
| 190 | DSG2 | Heterozygous | Pathogenic | c.146G>A | p.Arg49His |
| 180 | DSG2 | Heterozygous | Likely Pathogenic | c.1003A>G | p.Thr335Ala |
| 178 | DSG2 | Homozygous Recessive | Pathogenic | c.1003A>G | p.Thr335Ala |
| 208 | DSG2 | Heterozygous | Pathogenic | c.1003A>G | p.Thr335Ala |
| 270 | DSG2 | Heterozygous | Pathogenic | c.1038_1040delGAA | p.Lys346del |
| 206 | DSG2 | Heterozygous | Likely Pathogenic | c.1003A>G | p.Thr335Ala |
| 270 | DSG2 | Heterozygous | Pathogenic | c.1015delG | p.Glu339fsX340 |
| 79 | DSP | Heterozygous | Pathogenic | c.3416dupA | p.Tyr1139X |
| 80 | DSP | Heterozygous | Pathogenic | c.3475G>T | p.Glu1159X |
| 52 | DSP | Heterozygous | Pathogenic | c.5596C>T | p.Gln1866X |
| 201 | DSP | Heterozygous | Pathogenic | c.7699_7700insG | p.Val2567CysfsX14 |
| 237 | DSP | Heterozygous | Likely Pathogenic | c.3793G>T | p.Glu1265X |
| 50 | DSP | Heterozygous | Likely Pathogenic | c.3G>T | p.0? |
| 241 | DSP | Heterozygous | Pathogenic | c.2809_2810insAA | p.Ile937LysfsX49 |
| 200 | DSP | Heterozygous | Likely Pathogenic | c.7699_7700insG | p.Val2567CysfsX14 |
| 51 | DSP | Heterozygous | Likely Pathogenic | c.3505T>A | p.Tyr1169Asn |
| 78 | DSP | Heterozygous | Pathogenic | c.337C>T | p.Gln113X |
| 53 | DSP | Heterozygous | Pathogenic | c.5596C>T | p.Gln1866X |
| 54 | DSP | Heterozygous | Pathogenic | c.2821C>T | p.Arg941X |
| 55 | DSP | Heterozygous | Pathogenic | c.3741_3742insAAATCGA | p.Asp1248LysfsX7 |
| 75 | DSP | Heterozygous | Pathogenic | c.3298_3299insTGTT | p.Cys1100LeufsX11 |
| 56 | DSP | Heterozygous | Pathogenic | c.37_38delAC | p.Thr13SerfsX79 |
| 87 | DSP | Heterozygous | Pathogenic | c.7461_7464delAATT | p.Asp2489MetfsX17 |
| 57 | DSP | Heterozygous | Pathogenic | c.5851C>T | p.Arg1951X |
| 65 | DSP | Heterozygous | Pathogenic | c.540G>A | p.Trp180X |
| 62 | DSP | Heterozygous | Pathogenic | c.1288G>T | p.Glu430X |
| 61 | DSP | Heterozygous | Pathogenic | c.8469_8487delGGGGTCCCGCTCCGGCTCC | p.Ser2843_Arg2846del |
| 60 | DSP | Heterozygous | Pathogenic | c.8469_8487delGGGGTCCCGCTCCGGCTCC | p.Ser2843_Arg2846del |
| 66 | DSP | Heterozygous | Pathogenic | c.3928A>T | p.Lys1310X |
| 188 | DSP | Heterozygous | Pathogenic | c.5318delT | p.Leu1773TyrfsX8 |
| 175 | DSP | Heterozygous | Likely Pathogenic | c.5410C>T | p.Gln1804X |
| 212 | DSP | Heterozygous | Likely Pathogenic | c.5318delT | p.Leu1773TyrfsX8 |
| 211 | DSP | Heterozygous | Likely Pathogenic | c.5001delG | p.Gln1667HisfsX14 |
| 210 | DSP | Heterozygous | Likely Pathogenic | c.4672C>T | p.Arg1558Trp |
| 174 | DSP | Heterozygous | Likely Pathogenic | c.5410C>T | p.Gln1804X |
| 169 | DSP | Heterozygous | Likely Pathogenic deletion exons 21-23 | |  |
| 177 | DSP | Heterozygous | Likely Pathogenic deletion exons 21-23 | |  |
| 209 | DSP | Heterozygous | Likely Pathogenic | c.4672C>T | p.Arg1558Trp |
| 189 | DSP | Heterozygous | Pathogenic | c.5318delT | p.Leu1773TyrfsX8 |
| 48 | DSP | Heterozygous | Pathogenic | c.3928A>T | p.Lys1310X |
| 176 | DSP | Heterozygous | Pathogenic | c.5596C>T | p.Gln1866X |
| 183 | DSP | Heterozygous | Pathogenic | c.3115dupG | p.Glu1039GlyfsX15 |
| 185 | DSP | Heterozygous | Likely Pathogenic deletion exons 21-23 | |  |
| 186 | DSP | Heterozygous | Pathogenic | deletion including exon 21-23 |  |
| 187 | DSP | Heterozygous | Pathogenic | c.2956C>T | p.Gln986X |
| 179 | DSP | Heterozygous | Likely Pathogenic | c.1056_1061delCACTCT | p.Asp352_354delinsGlu |
| 145 | DSP | Heterozygous | Pathogenic | c.5773C>T | p.Gln1925X |
| 229 | DSP | Heterozygous | Likely Pathogenic | c.2390_2393delCAGA | p.Val797AlafsX14 |
| 227 | DSP | Heterozygous | Likely Pathogenic | c.170+5G>T |  |
| 92 | DSP | Heterozygous | Pathogenic | c.4457T>A | p.Leu1486X |
| 93 | DSP | Heterozygous | Pathogenic | c.4457T>A | p.Leu1486X |
| 223 | DSP | Heterozygous | Likely Pathogenic | c.2390_2393delCAGA | p.Val797AlafsX14 |
| 184 | DSP | Heterozygous | Pathogenic | c.3865C>T | p.Gln1289X |
| 119 | DSP | Heterozygous | Pathogenic | c.808C>T | p.Arg270X |
| 121 | DSP | Heterozygous | Pathogenic | c.808C>T | p.Arg270X |
| 182 | DSP | Heterozygous | Pathogenic | c.3865C>T | p.Gln1289X |
| 124 | DSP | Heterozygous | Pathogenic | c.540G>A | p.Trp180X |
| 136 | DSP | Heterozygous | Pathogenic | c.1273C>T | p.Arg425X |
| 180 | DSP | Heterozygous | Likely Pathogenic | c.8285A>C | p.Gln2762Pro |
| 232 | DSP | Heterozygous | Likely Pathogenic | c.6393delA | p.Gly2133VfsX2 |
| 1 | DSP | Heterozygous | Pathogenic | c.3337C>T | p.Arg1113X |
| 46 | DSP | Heterozygous | Likely Pathogenic | c.1267-1A>G |  |
| 252 | DSP | Heterozygous | Likely Pathogenic | c.1520C>T | p.Ser507Phe |
| 253 | DSP | Heterozygous | Likely Pathogenic | c.1520C>T | p.Ser507Phe |
| 260 | DSP | Heterozygous | Pathogenic | c.1873C>T | p.Gln625X |
| 265 | DSP | Heterozygous | Pathogenic | c.2821C>T | p.Arg941X |
| 267 | DSP | Heterozygous | Pathogenic | c.926_927insG | p.Gln309fsX15 |
| 269 | DSP | Heterozygous | Pathogenic | c.8077_8080delAGAA | p.Lys2693ProfsX3 |
| 271 | DSP | Heterozygous | Pathogenic | c.8188delC | p.Gln2730SerfsX16 |
| 275 | DSP | Heterozygous | Pathogenic | c.4477G>T | p.Glu1493X |
| 276 | DSP | Heterozygous | Pathogenic | c.4477G>T | p.Glu1493X |
| 14 | DSP | Heterozygous | Pathogenic | c.939+1G>C |  |
| 288 | DSP | Heterozygous | Pathogenic | c.5659_5660delAA | p.Glu1886LysfsX3 |
| 290 | DSP | Heterozygous | Pathogenic | c.1754_1755insA | p.His586ThrfsX9 |
| 2 | DSP | Heterozygous | Pathogenic | c.1754_1755insA | p.His586ThrfsX9 |
| 10 | DSP | Heterozygous | Pathogenic | c.2958G>T | p.Gln986His |
| 5 | DSP | Heterozygous | Pathogenic | c.3045delG | p.Arg1015SerfsX3 |
| 6 | DSP | Heterozygous | Pathogenic | c.3045delG | p.Arg1015SerfsX3 |
| 245 | DSP | Heterozygous | Pathogenic | c.3045delG | p.Arg1015SerfsX3 |
| 41 | DSP | Heterozygous | Pathogenic | c.6273delA | p.Ala2092LeufsX24 |
| 40 | DSP | Heterozygous | Pathogenic | c.6273delA | p.Ala2092LeufsX24 |
| 26 | DSP | Heterozygous | Likely Pathogenic | c.1520C>T | p.Ser507Phe |
| 49 | DSP | Heterozygous | Pathogenic | c.1831C>T | p.Gln611X |
| 69 | DSP | Heterozygous | Pathogenic | c.1754_1755insA | p.His586ThrfsX9 |
| 42 | DSP | Heterozygous | Pathogenic | c.8077_8080delAAAG | p.Lys2693ProfsX3 |
| 22 | DSP | Heterozygous | Likely Pathogenic | c.1520C>T | p.Ser507Phe |
| 35 | DSP | Heterozygous | Pathogenic | c.305C>T | p.Arg1269X |
| 44 | DSP | Heterozygous | Pathogenic | c.939+1G>A |  |
| 45 | DSP | Heterozygous | Likely Pathogenic | c.1267-1A>G |  |
| 43 | DSP | Heterozygous | Pathogenic | c.939+1G>A |  |
| 3 | FLNC | Heterozygous | Pathogenic | c.2971C>T | p.Arg991X |
| 4 | FLNC | Heterozygous | Pathogenic | c.2971C>T | p.Arg991X |
| 242 | FLNC | Heterozygous | Pathogenic | c.2234delG | p.Gly745GlufsX3 |
| 228 | FLNC | Heterozygous | Likely Pathogenic | c.7251 +1G>A |  |
| 72 | FLNC | Heterozygous | Pathogenic | c.2971C>T | p.Arg991X |
| 224 | FLNC | Heterozygous | Likely Pathogenic | c.6565G>T | p.Glu2189X |
| 172 | FLNC | Heterozygous | Pathogenic | c.5395C>T | p.Gly1800X |
| 64 | FLNC | Heterozygous | Pathogenic | c.1444C>T | p.Arg482X |
| 231 | FLNC | Heterozygous | Likely Pathogenic | c.6565G>T | p.Glu2189X |
| 63 | FLNC | Heterozygous | Pathogenic | c.1444C>T | p.Arg482X |
| 105 | JUP | Homozygous Recessive | Pathogenic | c.2040_2041delGT ( 2157del2) | p.Gly680fsX690 |
| 113 | JUP | Homozygous Recessive | Pathogenic | c.2040_2041delGT ( 2157del2) | p.Gly680fsX690 |
| 112 | JUP | Homozygous Recessive | Pathogenic | c.2040_2041delGT ( 2157del2) | p.Gly680fsX690 |
| 111 | JUP | Homozygous Recessive | Pathogenic | c.2040_2041delGT ( 2157del2) | p.Gly680fsX690 |
| 109 | JUP | Homozygous Recessive | Pathogenic | c.2040_2041delGT ( 2157del2) | p.Gly680fsX690 |
| 108 | JUP | Homozygous Recessive | Pathogenic | c.2040_2041delGT ( 2157del2) | p.Gly680fsX690 |
| 107 | JUP | Homozygous Recessive | Pathogenic | c.2040_2041delGT ( 2157del2) | p.Gly680fsX690 |
| 106 | JUP | Homozygous Recessive | Pathogenic | c.2040_2041delGT ( 2157del2) | p.Gly680fsX690 |
| 104 | JUP | Homozygous Recessive | Pathogenic | c.2040_2041delGT ( 2157del2) | p.Gly680fsX690 |
| 100 | JUP | Homozygous Recessive | Pathogenic | c.2040_2041delGT ( 2157del2) | p.Gly680fsX690 |
| 102 | JUP | Homozygous Recessive | Pathogenic | c.2040_2041delGT ( 2157del2) | p.Gly680fsX690 |
| 99 | JUP | Homozygous Recessive | Pathogenic | c.2040_2041delGT ( 2157del2) | p.Gly680fsX690 |
| 98 | JUP | Homozygous Recessive | Pathogenic | c.2040_2041delGT ( 2157del2) | p.Gly680fsX690 |
| 97 | JUP | Homozygous Recessive | Pathogenic | c.2040_2041delGT ( 2157del2) | p.Gly680fsX690 |
| 96 | JUP | Homozygous Recessive | Pathogenic | c.2040_2041delGT ( 2157del2) | p.Gly680fsX690 |
| 95 | JUP | Homozygous Recessive | Pathogenic | c.2040_2041delGT ( 2157del2) | p.Gly680fsX690 |
| 128 | JUP | Homozygous Recessive | Pathogenic | c.2040_2041delGT ( 2157del2) | p.Gly680fsX690 |
| 103 | JUP | Homozygous Recessive | Pathogenic | c.2040_2041delGT ( 2157del2) | p.Gly680fsX690 |
| 125 | JUP | Homozygous Recessive | Pathogenic | c.2040_2041delGT ( 2157del2) | p.Gly680fsX690 |
| 126 | JUP | Homozygous Recessive | Pathogenic | c.2040_2041delGT ( 2157del2) | p.Gly680fsX690 |
| 127 | JUP | Homozygous Recessive | Pathogenic | c.2040_2041delGT ( 2157del2) | p.Gly680fsX690 |
| 257 | PKP2 | Heterozygous | Pathogenic | c.1799delA | p.Asp600ValfsX56 |
| 218 | PKP2 | Heterozygous | Pathogenic | c.1643delG | p.Gly548ValfsX15 |
| 219 | PKP2 | Heterozygous | Pathogenic | c.775_776insG | p.Glu259GlyfsX77 |
| 221 | PKP2 | Heterozygous | Pathogenic | c.1643delG | p.Gly548ValfsX15 |
| 194 | PKP2 | Heterozygous | Pathogenic | c.1643delG | p.Gly548ValfsX15 |
| 195 | PKP2 | Heterozygous | Pathogenic | c.775_776insG | p.Glu259GlyfsX77 |
| 196 | PKP2 | Heterozygous | Pathogenic | c.775_776insG | p.Glu259GlyfsX77 |
| 236 | PKP2 | Heterozygous | Likely Pathogenic | c.1171-7307_21341delinsATAATG. p.Val391-882del | |
| 235 | PKP2 | Heterozygous | Likely Pathogenic | c.419C>T | p.Ser140Phe |
| 197 | PKP2 | Heterozygous | Pathogenic | c.775_776insG | p.Glu259GlyfsX77 |
| 234 | PKP2 | Heterozygous | Likely Pathogenic | c.964_965delGG | p.Gly322ProfsX13 |
| 198 | PKP2 | Heterozygous | Pathogenic | c.775_776insG | p.Glu259GlyfsX77 |
| 204 | PKP2 | Heterozygous | Pathogenic | c.148_151delACAG | p.Thr50SerfsX61 |
| 199 | PKP2 | Heterozygous | Likely Pathogenic | c.983delG | p.Gly328GlufsX24 |
| 192 | PKP2 | Heterozygous | Likely Pathogenic | c.983delG | p.Gly328GlufsX24 |
| 264 | PKP2 | Heterozygous | Pathogenic | c.1799delA | p.Asp600ValfsX56 |
| 27 | PKP2 | Heterozygous | Pathogenic | c.2146-G>C |  |
| 263 | PKP2 | Heterozygous | Pathogenic | c.2489+1G>A |  |
| 262 | PKP2 | Heterozygous | Pathogenic | c.1799delA | p.Asp600ValfsX56 |
| 10 | PKP2 | Heterozygous | Pathogenic | c.1844C>T | p.Ser615Phe |
| 181 | PKP2 | Heterozygous | Likely Pathogenic | 2578-2A>C |  |
| 261 | PKP2 | Heterozygous | Pathogenic | c.2062T>C | p.Ser688Pro |
| 28 | PKP2 | Heterozygous | Pathogenic | c.223G>T | p.Gly75X |
| 240 | PKP2 | Heterozygous | Pathogenic | c.2146-1G>C |  |
| 259 | PKP2 | Heterozygous | Pathogenic | c.1844C>T | p.Ser615Phe |
| 77 | PKP2 | Heterozygous | Pathogenic | c.175C>T | p.Gln59X |
| 258 | PKP2 | Heterozygous | Pathogenic | c.1799delA | p.Asp600ValfsX56 |
| 191 | PKP2 | Heterozygous | Pathogenic | c.148_151delACAG | p.Thr50SerfsX61 |
| 76 | PKP2 | Heterozygous | Pathogenic | c.2013delC | p.Lys672ArgfsX12 |
| 29 | PKP2 | Heterozygous | Pathogenic | c.223G>T | p.Gly75X |
| 74 | PKP2 | Heterozygous | Pathogenic | c.148_151delACAG | p.Thr50SerfsX61 |
| 32 | PKP2 | Heterozygous | Pathogenic | c.2489+1G>A |  |
| 70 | PKP2 | Heterozygous | Pathogenic | c.2146-1G>C |  |
| 222 | PKP2 | Heterozygous | Pathogenic | c.1514delT | p.Leu505CysfsX15 |
| 86 | PKP2 | Heterozygous | Pathogenic | c.1643delG | p.Gly548ValfsX5 |
| 67 | PKP2 | Homozygous Recessive | Likely Pathogenic | c.1941T>G | p.Cys647Trp |
| 217 | PKP2 | Heterozygous | Likely Pathogenic | c.775_776insG | p.Glu259GlyfsX77 |
| 38 | PKP2 | Heterozygous | Pathogenic | c.2197_2202delinsG | p.His733AlafsX8 |
| 36 | PKP2 | Heterozygous | Pathogenic | c.337-2A>T |  |
| 230 | PKP2 | Heterozygous | Likely Pathogenic | c.1171_2646del | p.Val391_882del |
| 225 | PKP2 | Heterozygous | Likely Pathogenic | c.1171-7307_*21341delinsATAATG |  |
| 226 | PKP2 | Heterozygous | Likely Pathogenic | c.1264_1265delTT | p.Leu422SerfsX3 |
| 34 | PKP2 | Heterozygous | Pathogenic | c.2197_2202delinsG | p.His733AlafsX8 |
| 37 | PKP2 | Heterozygous | Pathogenic | c.808C>T | p.Gln270X |
| 58 | PKP2 | Heterozygous | Pathogenic | c.2027G>A | p.Trp676X |
| 216 | PKP2 | Heterozygous | Pathogenic | c.775_776insG | p.Glu259GlyfsX77 |
| 30 | PKP2 | Heterozygous | Pathogenic | c.1237C>T | p.Arg413X |
| 250 | PKP2 | Heterozygous | Pathogenic | c.1237C>T | p.Arg413X |
| 249 | PKP2 | Heterozygous | Pathogenic | c.2146-1G>C |  |
| 248 | PKP2 | Heterozygous | Pathogenic | c.2489+1G>A |  |
| 247 | PKP2 | Heterozygous | Pathogenic | c.148_151delACAG | p.Thr50SerfsX61 |
| 31 | PKP2 | Heterozygous | Pathogenic | c.2478delT | p.Ser826Argfs |
| 215 | PKP2 | Heterozygous | Likely Pathogenic | c.775_776insG | p.Glu259GlyfsX77 |
| 88 | PKP2 | Heterozygous | Pathogenic | c.2013delC | p.Lys672ArgfsX12 |
| 243 | PKP2 | Heterozygous | Pathogenic | c.1643delG | p.Gly548ValfsX5 |
| 39 | PKP2 | Heterozygous | Likely Pathogenic | Not available | p.Glu624fs |
| 213 | PKP2 | Heterozygous | Likely Pathogenic | c.1171_1510del | p.Asn392CysfsX15 |
| 135 | PKP2 | Heterozygous | Pathogenic | c.2146-1G>C |  |
| 114 | PKP2 | Heterozygous | Pathogenic | c.1138G>T | p.Glu380X |
| 9 | PKP2 | Heterozygous | Pathogenic | c.2489+1G>A |  |
| 149 | PKP2 | Heterozygous | Pathogenic | c.971_980delCCTGCCGCCGCCTG | p.Ala324GlyfsX25 |
| 142 | PKP2 | Heterozygous | Likely Pathogenic | IVS1+1G>A |  |
| 143 | PKP2 | Heterozygous | Pathogenic | c.275T>A | p.Leu92X |
| 286 | PKP2 | Heterozygous | Pathogenic | c.1237C>T | p.Arg413X |
| 12 | PKP2 | Heterozygous | Pathogenic | c.2146-1G>C |  |
| 285 | PKP2 | Heterozygous | Pathogenic | c.2197_2202delinsG | p.His733AlafsX8 |
| 146 | PKP2 | Heterozygous | Pathogenic | c.275T>A | p.Leu92X |
| 284 | PKP2 | Heterozygous | Pathogenic | c.2197_2202delinsG | p.His733AlafsX8 |
| 282 | PKP2 | Heterozygous | Pathogenic | c.2489+1G>T | |
| 141 | PKP2 | Heterozygous | Likely Pathogenic | IVS1+1G>A |  |
| 281 | PKP2 | Heterozygous | Pathogenic | c.2489+1G>T | |
| 150 | PKP2 | Heterozygous | Pathogenic | c.971_980delCCTGCCGCCGCCTG | p.Ala324GlyfsX25 |
| 156 | PKP2 | Heterozygous | Pathogenic | c.1643delG | p.Gly548ValfsX15 |
| 13 | PKP2 | Heterozygous | Pathogenic | c.2489+1G>A |  |
| 19 | PKP2 | Heterozygous | Pathogenic | c.1613G>A | p.Trp538X |
| 280 | PKP2 | Heterozygous | Pathogenic | c.2489+1G>A | |
| 20 | PKP2 | Heterozygous | Pathogenic | c.1613G>A | p.Trp538X |
| 154 | PKP2 | Heterozygous | Pathogenic | c.1643delG | p.Gly548ValfsX15 |
| 153 | PKP2 | Heterozygous | Pathogenic | c.971_980delCCTGCCGCCGCCTG | p.Ala324GlyfsX25 |
| 140 | PKP2 | Heterozygous | Likely Pathogenic | IVS1+1G>A |  |
| 287 | PKP2 | Heterozygous | Pathogenic | c.2197_2202delinsG | p.His733AlafsX8 |
| 147 | PKP2 | Heterozygous | Pathogenic | c.1063C>T | p.Arg355X |
| 8 | PKP2 | Heterozygous | Pathogenic | c.148_151delACAG | p.Thr50SerfsX61 |
| 122 | PKP2 | Heterozygous | Pathogenic | c.1138G>T | p.Glu380X |
| 123 | PKP2 | Heterozygous | Pathogenic | c.1138G>T | p.Glu380X |
| 117 | PKP2 | Heterozygous | Pathogenic | c.971_980delCCTGCCGCCGCCTG | p.Ala324GlyfsX25 |
| 129 | PKP2 | Heterozygous | Pathogenic | c.1202_1209delTGCAGCTC | p.Leu401ProfsX6 |
| 130 | PKP2 | Heterozygous | Pathogenic | c.1202_1209delTGCAGCTC | p.Leu401ProfsX6 |
| 131 | PKP2 | Heterozygous | Pathogenic | c.1689-?_1806+?del | |
| 116 | PKP2 | Heterozygous | Pathogenic | c.971_980delCCTGCCGCCGCCTG | p.Ala324GlyfsX25 |
| 132 | PKP2 | Heterozygous | Pathogenic | c.1689-?_1806+?del | |
| 115 | PKP2 | Heterozygous | Pathogenic | c.1138G>T | p.Glu380X |
| 110 | PKP2 | Heterozygous | Pathogenic | c.971_980delCCTGCCGCCGCCTG | p.Ala324GlyfsX25 |
| 101 | PKP2 | Heterozygous | Pathogenic | c.971_980delCCTGCCGCCGCCTG | p.Ala324GlyfsX25 |
| 94 | PKP2 | Heterozygous | Pathogenic | c.2146-1G>C |  |
| 289 | PKP2 | Heterozygous | Pathogenic | c.2146-1G>C |  |
| 11 | PKP2 | Heterozygous | Pathogenic | c.1844C>T | p.Ser615Phe |
| 155 | PKP2 | Heterozygous | Pathogenic | c.1643delG | p.Gly548ValfsX15 |
| 279 | PKP2 | Heterozygous | Pathogenic | c.582T>G | p.Tyr194X |
| 160 | PKP2 | Heterozygous | Pathogenic | c.987delT | p.Ser329ArgfsX23 |
| 162 | PKP2 | Heterozygous | Pathogenic | c.987delT | p.Ser329ArgfsX23 |
| 274 | PKP2 | Heterozygous | Pathogenic | c.2197_2202delinsG | p.His733AlafsX8 |
| 85 | PKP2 | Heterozygous | Pathogenic | c.148_151delACAG | p.Thr50SerfsX61 |
| 21 | PKP2 | Heterozygous | Pathogenic | c.1613G>A | p.Trp538X |
| 277 | PKP2 | Heterozygous | Likely Pathogenic | c.419C>T | p.Ser140Phe |
| 166 | PKP2 | Heterozygous | Pathogenic | c.987delT | p.Ser329ArgfsX23 |
| 165 | PKP2 | Heterozygous | Pathogenic | c.987delT | p.Ser329ArgfsX23 |
| 272 | PKP2 | Heterozygous | Pathogenic | c.235C>T | p.Arg79X |
| 164 | PKP2 | Heterozygous | Pathogenic | c.987delT | p.Ser329ArgfsX23 |
| 163 | PKP2 | Heterozygous | Pathogenic | c.987delT | p.Ser329ArgfsX23 |
| 269 | PKP2 | Heterozygous | Pathogenic | c.2146-1G>C | |
| 268 | PKP2 | Heterozygous | Pathogenic | c.2197_2202delinsG | p.His733AlafsX8 |
| 170 | PKP2 | Heterozygous | Likely Pathogenic | c.2578G>T | p.Ala860Ser |
| 273 | PKP2 | Heterozygous | Pathogenic | c.2197_2202delinsG | p.His733AlafsX8 |
| 173 | PKP2 | Heterozygous | Likely Pathogenic | c.1799delA | p.Asp600ValfsX56 |
| 171 | PKP2 | Heterozygous | Likely Pathogenic | c.14delG | p.Gly5TrpfsX38 |
| 157 | PKP2 | Heterozygous | Pathogenic | c.1643del | p.Gly548ValfsX15 |
| 47 | PKP2 | Heterozygous | Pathogenic | c.2490-35_*3043del | p.Ala830fs |
| 23 | PKP2 | Heterozygous | Pathogenic | c.2489+1G>A |  |
| 25 | PKP2 | Heterozygous | Pathogenic | c.2489+1G>A |  |
| 158 | PKP2 | Heterozygous | Pathogenic | c.2489+1G>A |  |
| 266 | PKP2 | Heterozygous | Pathogenic | c.2146-1G>C | |
| 24 | PKP2 | Heterozygous | Pathogenic | c.2489+1G>A |  |
| 161 | PKP2 | Heterozygous | Pathogenic | c.987delT | p.Ser329ArgfsX23 |
| 159 | PKP2 | Heterozygous | Pathogenic | c.987delT | p.Ser329ArgfsX23 |
| 137 | PLN | Heterozygous | Pathogenic | c.40_42delAGA | p.Arg14del |
| 138 | PLN | Heterozygous | Pathogenic | c.40_42delAGA | p.Arg14del |
| 139 | PLN | Heterozygous | Pathogenic | c.40_42delAGA | p.Arg14del |
| 256 | RBM20 | Heterozygous | Pathogenic | c.1913C>T | p.Pro638Leu |
| 167 | TMEM43 | Heterozygous | Pathogenic | c.1073C>T | p.Ser358Leu |
| 168 | TMEM43 | Heterozygous | Pathogenic | c.1073C>T | p.Ser358Leu |

**Supplementary Table S4:** Sensitivity analyses of key findings in model validation metrics between different datasets and subgroups.

| **Metric** | **Complete-case dataset** | **CMR dataset** | **Final dataset used** |
| --- | --- | --- | --- |
| Full cohort | n=266  events=34  Uno’s 0.73  (95% CI 0.63-0.82)  Slope 0.43  (95% CI 0.18-0.70) | n=359  events=53  Uno’s 0.74  (95% CI 0.67-0.81)  Slope 0.48  (95% CI 0.30-0.75) | n=554  events=100  Uno’s 0.75  (95% CI 0.70-0.81)  Slope 0.52  (95% CI 0.37-0.71) |
| Gene positive cohort | n=140  events=22  Uno’s 0.83  (95% CI 0.74-0.92)  Slope 0.75  (95% CI 0.37-1.15) | n=196  events=31  Uno’s 0.82  (95% CI 0.74-0.89)  Slope 0.66  (95% CI 0.37-1.34) | n=290*  events=56  Uno’s 0.82  (95% CI 0.76-0.88)  Slope 0.78  (95% CI 0.53-1.06) |
| PKP2 cohort | n=56  events=15  Uno’s 0.81  (95% CI 0.71-0.93)  Slope 1.12  (95% CI 0.67-1.82) | n=79  events=17  Uno’s 0.80  (95% CI 0.70-0.92)  Slope 0.77  (95% CI 0.37-1.34) | n=118  events=27  Uno’s 0.83  (95% CI 0.75-0.91)  Slope 0.73  (95% CI 0.44-1.14) |
| Gene elusive cohort | n=91  events=8  Uno’s 0.60  (95% CI 0.40-0.82)  Slope 0.13  (95% CI -0.19-0.50) | n=149  events=22  Uno’s 0.65  (95% CI 0.53-0.77)  Slope 0.39  (95% CI 0.06-0.74) | n=234*  events=38  Uno’s 0.65  (95% CI 0.57-0.74)  Slope 0.27  (95% CI 0.06-0.55) |

* Patients with missing genetic data were assigned to gene positive or gene elusive as per the multiple imputation with chained equations procedure. A total of 104 cases were re-classified.

# Supplementary Figure Legends

**Supplementary Figure 1:** Nullity matrix for all variables that were used in the imputation procedure. CMR=cardiac magnetic resonance; LGE=late gadolinium enhancement; LV=left ventricular; LVEDV=left ventricular end-diastolic volume; LVEF=left ventricular ejection fraction; MICE=multivariate imputation with chained equations; PVCs=premature ventricular contractions; RV=right ventricular; RVEDV=right ventricular end-diastolic volume; RVEF=right ventricular ejection fraction; VT=ventricular tachycardia.

**Supplementary Figure 2**: Kernel density estimation (KDE) plots of each of the imputed variables and the resulting corrected 5-year ARVC risk score for each imputation model and the complete-case dataset are shown. Model 5 was selected as it demonstrated the best resulting probability density of the RVEF variable.

**Supplementary Figure 3**: Histogram demonstrating the distribution of the corrected 5-year ARVC risk score in the ‘validation’ dataset. Kernel density estimation is plotted as a blue line.

**Supplementary Figure 4**: Cumulative incidence curves for VA stratified by genotype among the gene-positive cohort. 95% Confidence intervals are not shown for visual simplicity.

**Supplementary Figure 5**: Cumulative incidence curves for VA stratified by sex among the different gene groups.

**Supplementary Figure 6**: Calibration plots and respective calibration metrics for the full cohort gene groups according to the original 5-year 2019 ARVC risk score.
