# Supplementary figures and images for: Importance of genotype for risk stratification in arrhythmogenic right ventricular cardiomyopathy using the 2019 ARVC risk calculator
Source: Eur Heart J. 2022 Jun 29;43(32):3053–67. doi: 10.1093/eurheartj/ehac235 (PMC9392652; doi:10.1093/eurheartj/ehac235)

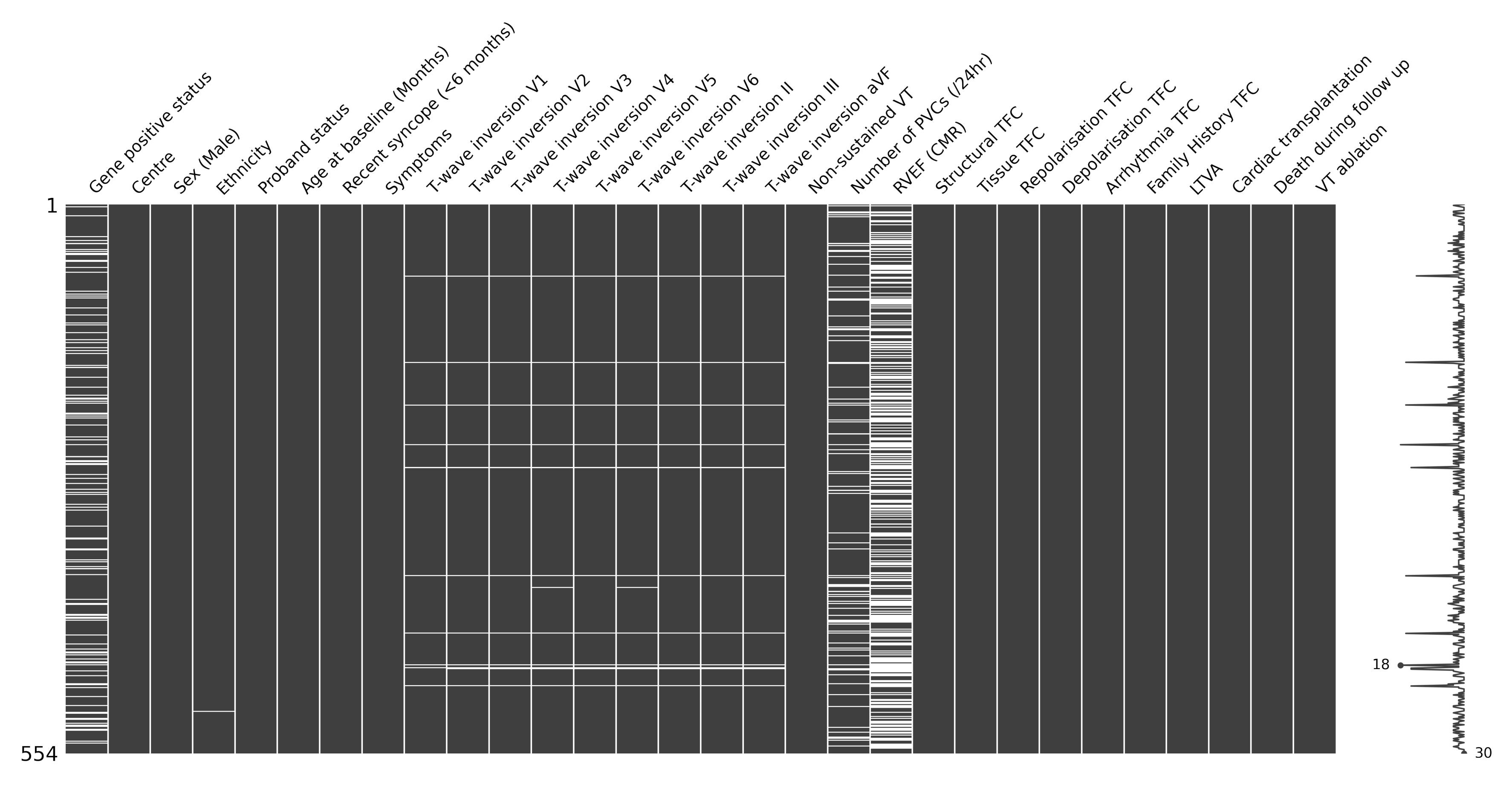

Supplement: ehac235_Supplementary_Data [file ehac235_supplementary_data.zip › Supplementary figure 1.tiff]

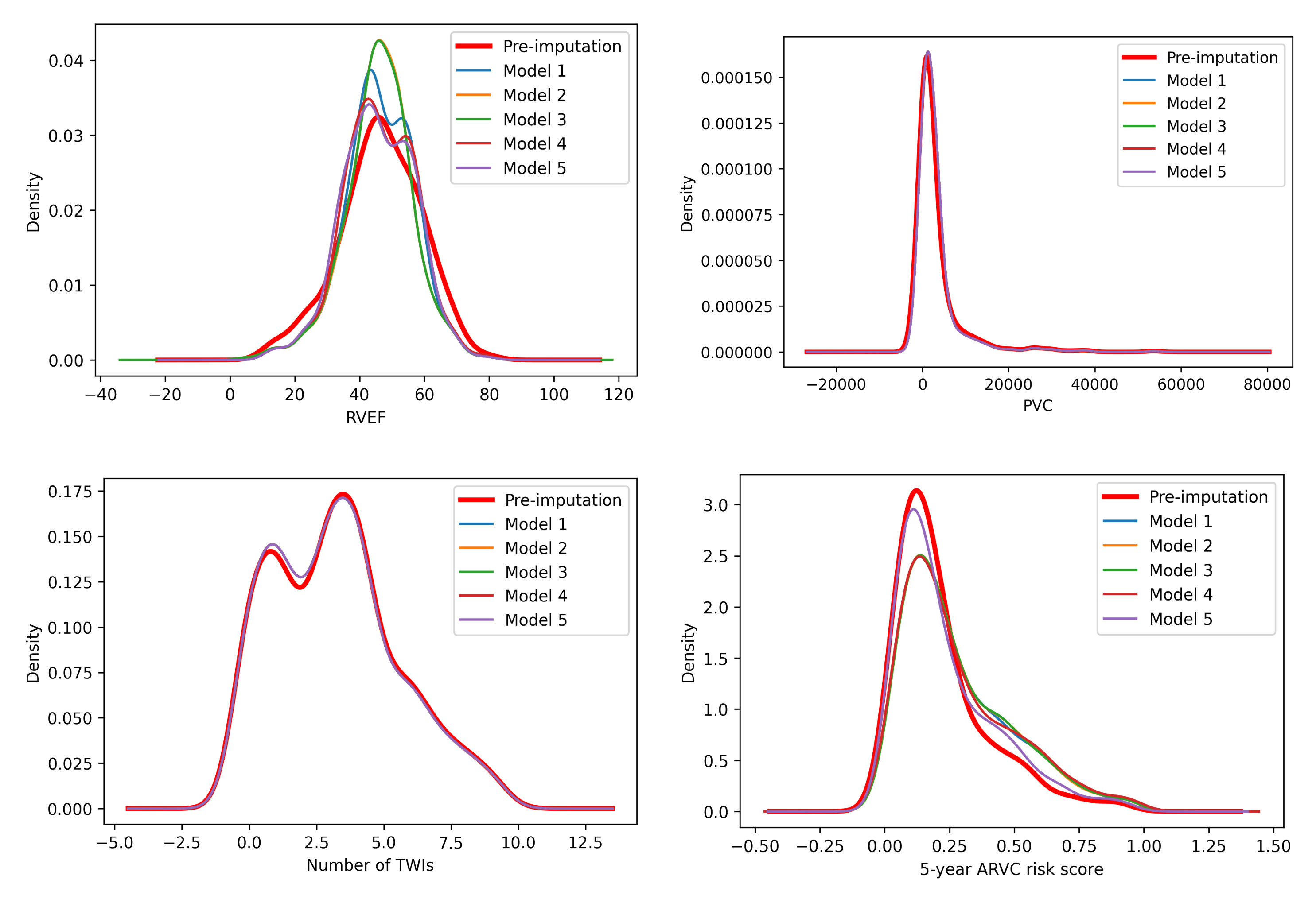

Supplement: ehac235_Supplementary_Data [file ehac235_supplementary_data.zip › Supplementary Figure 2.tiff]

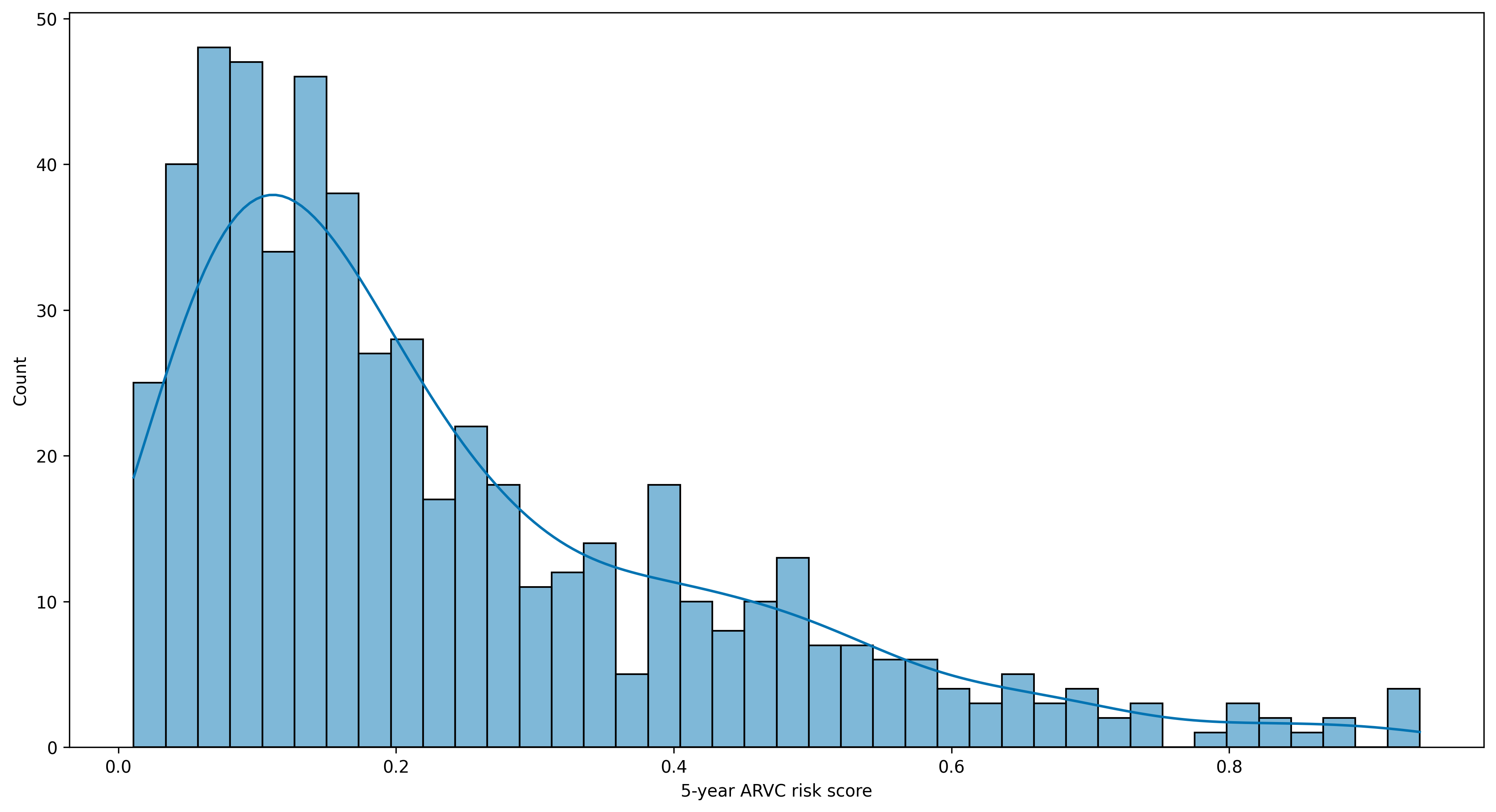

Supplement: ehac235_Supplementary_Data [file ehac235_supplementary_data.zip › Supplementary figure 3.tiff]

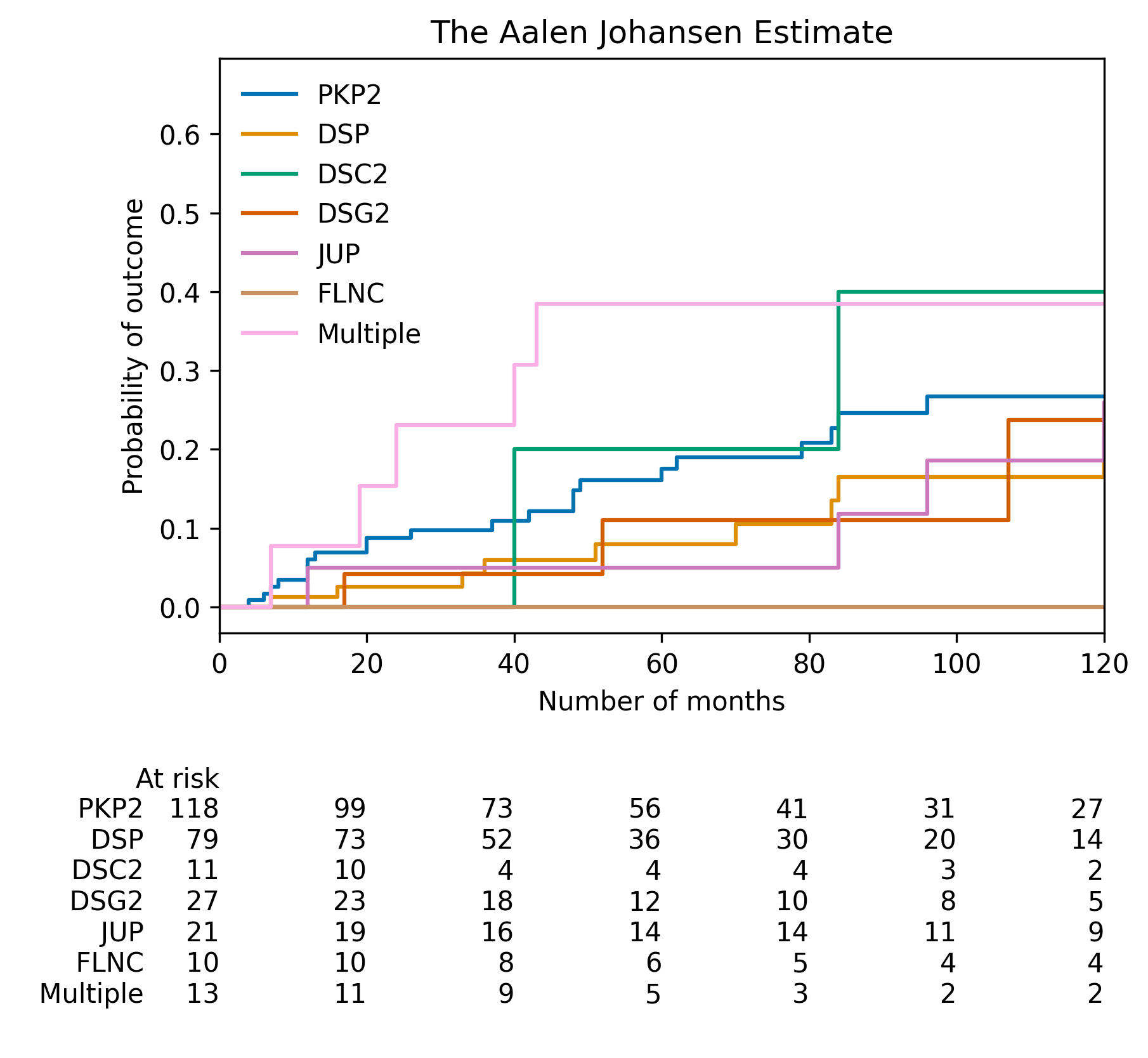

Supplement: ehac235_Supplementary_Data [file ehac235_supplementary_data.zip › Supplementary Figure 4.tiff]

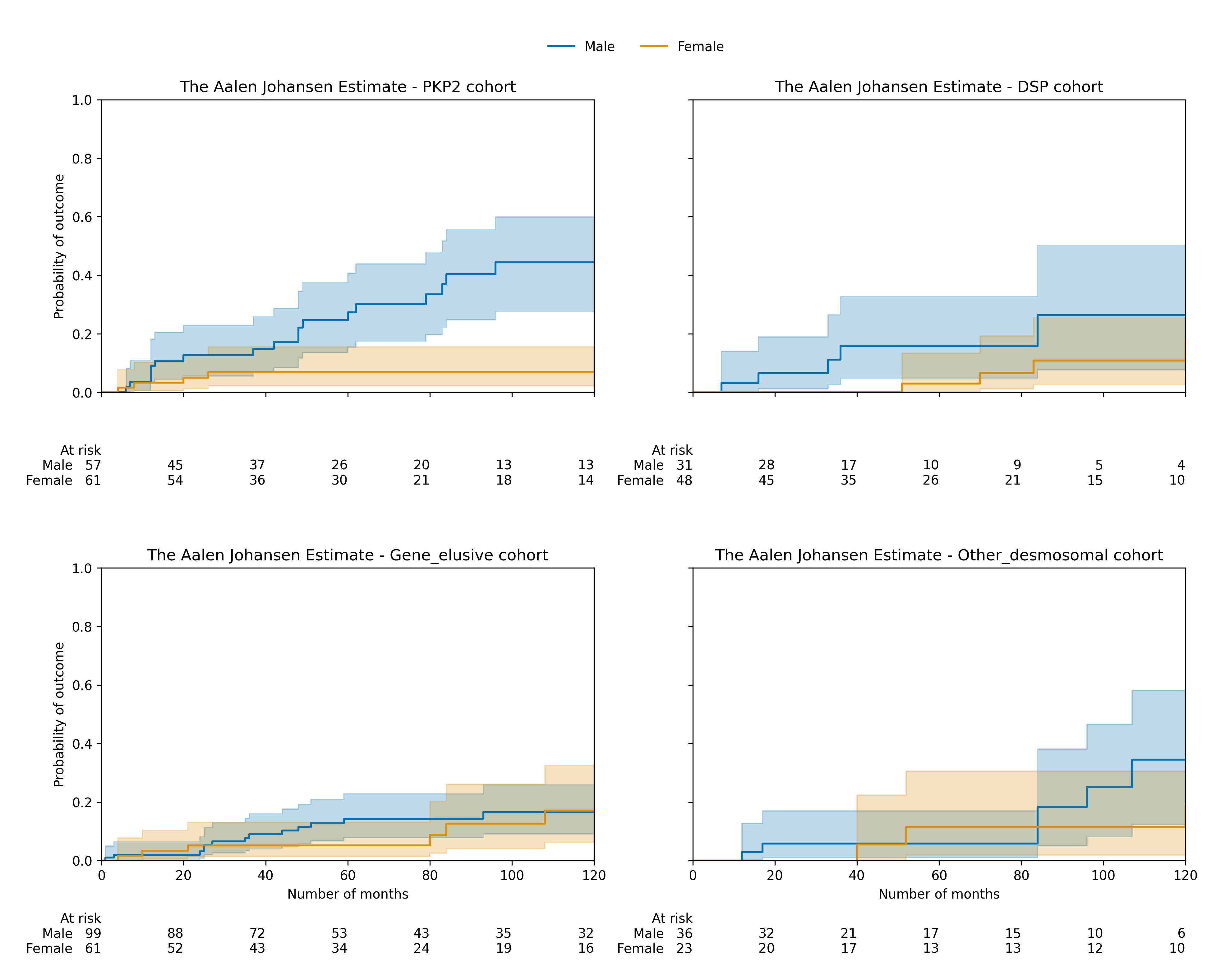

Supplement: ehac235_Supplementary_Data [file ehac235_supplementary_data.zip › Supplementary Figure 5.tiff]

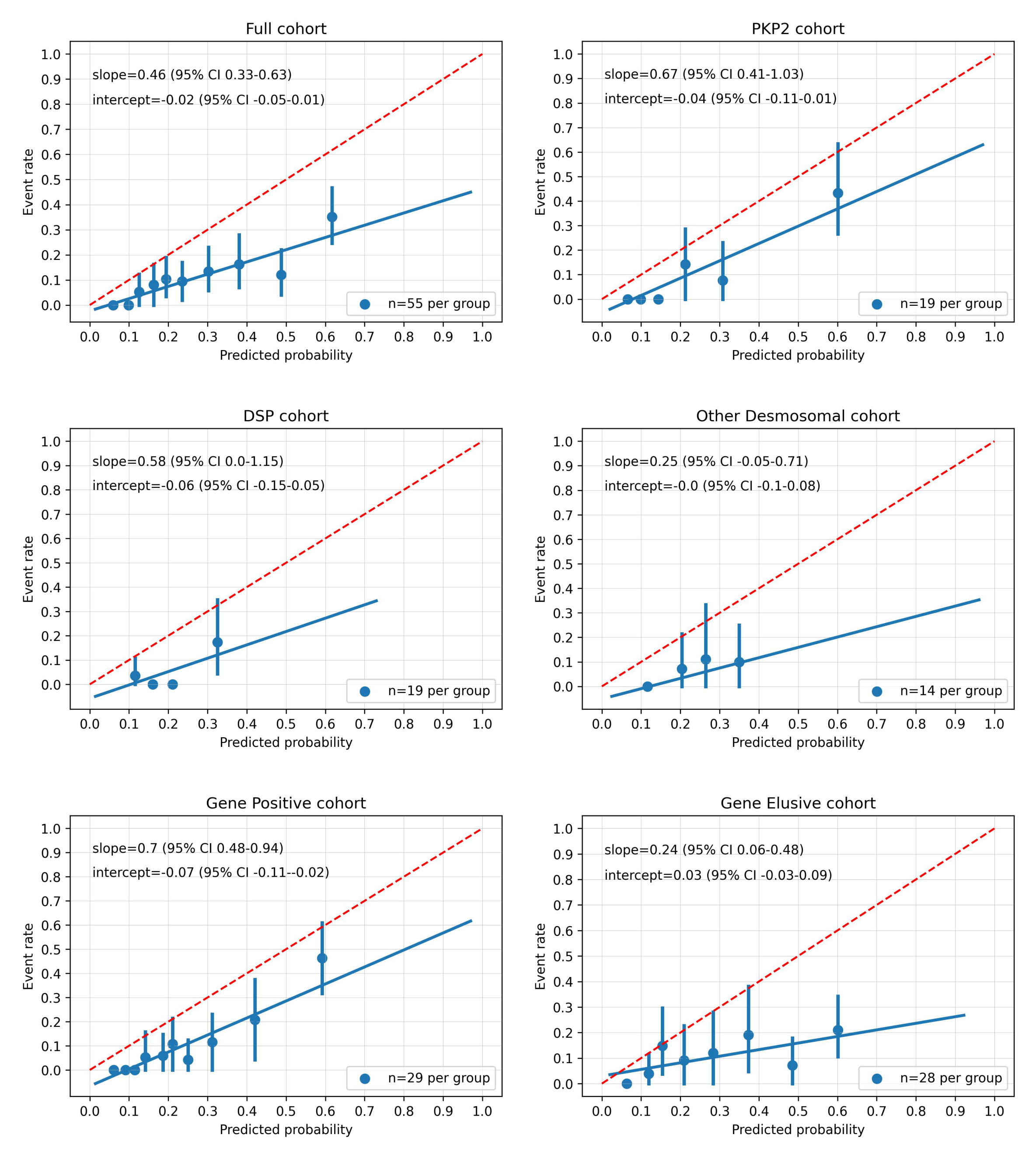

Supplement: ehac235_Supplementary_Data [file ehac235_supplementary_data.zip › Supplementary Figure 6.tiff]
